# Mice Born to Mothers Fed a Diet High in Omega-6 Fatty Acids and Low in Omega-3 Fatty Acids During Pregnancy Exhibit Various Behavioral Changes Including Impaired Social Behaviors and Enhanced Recognition Memory

**DOI:** 10.1016/j.tjnut.2024.12.031

**Published:** 2025-01-03

**Authors:** Nobuyuki Sakayori, Kazuki Fujii, Masanori Katakura, Mayumi Adachi, Yumie Koshidaka, Keizo Takao, Makoto Sugita

**Affiliations:** 1 Department of Physiology and Oral Physiology, Graduate School of Biomedical and Health Sciences, Hiroshima University, Hiroshima, Japan; 2 Department of Behavioral Physiology, Faculty of Medicine, University of Toyama, Toyama, Japan; 3 Life Science Research Center, University of Toyama, Toyama, Japan; 4 Research Center for Idling Brain Science, University of Toyama, Toyama, Japan; 5 Laboratory of Nutritional Physiology, Department of Pharmaceutical Sciences, Faculty of Pharmacy and Pharmaceutical Sciences, Josai University, Saitama, Japan

**Keywords:** essential fatty acids, arachidonic acid, docosapentaenoic acid, docosahexaenoic acid, prenatal, preterm, development, brain, motor coordination, fear memory

## Abstract

**Background:**

Modern dietary trends have led to an increase in foods that are relatively high in *n*–6 PUFAs and low in *n*–3 PUFAs. We previously reported that the offspring of mother mice that consumed a diet high in *n*–6 linoleic acid (LA) and low in *n*–3 α-linolenic acid (ALA), hereinafter called the LA^high^/ALA^low^ diet, exhibited behavioral abnormalities related to anxiety and feeding.

**Objectives:**

We currently lack a comprehensive overview of the behavioral abnormalities in these offspring, which was investigated in this study.

**Methods:**

C57BL/6J virgin female mice at 11 wk of age were fed either a control diet or the LA^high^/ALA^low^ diet, mated at 13 wk of age, and maintained on their respective diet throughout gestation. At birth, the lactating mothers’ diet was replaced with standard laboratory feed pellets. After weaning, the offspring continued to receive standard laboratory feed pellets, and both male and female offspring at 1–63 wk of age were analyzed using a comprehensive behavioral test battery (*n* = 6–14 offspring/group and offspring in each group were derived from ≥3 independent litters).

**Results:**

Both male and female offspring exposed in utero to the LA^high^/ALA^low^ diet exhibited impaired social behaviors, including the lower number of contacts with novel mice in the social interaction test [diet, *F*_(1,15)_ = 9.807, *P* = 0.007, 2-way analysis of variance (ANOVA)], and also showed enhanced recognition memory in the object location test (diet, *F*_(1,36)_ = 6.779, *P* = 0.013, 2-way ANOVA) compared with offspring exposed in utero to the control diet. In addition, compared with sex-matched controls, female offspring displayed hyperactivity in the open field test (*F*_(1,36)_ = 6.097, *P* = 0.018, simple main effect analysis).

**Conclusions:**

The maternal balance between dietary *n*–6 and *n*–3 PUFAs during pregnancy can have significant effects on the offspring’s behaviors, lasting well into adulthood.

## Introduction

Both *n*–6 and *n*–3 PUFAs are essential nutrients, as most species of the animal cannot synthesize these PUFAs de novo and must, therefore, obtain them from dietary sources. In the brain, the principal *n*–6 and *n*–3 PUFAs are arachidonic acid (ARA) and DHA, respectively [1], whereas linoleic acid (LA) and α-linolenic acid (ALA) are the principal *n*–6 and *n*–3 PUFAs in a diet, respectively [2,3], with ARA and DHA believed to be synthesized from dietary LA and ALA, respectively. Importantly, ARA and DHA have many enzymes in common with respect to their synthesis from LA and ALA, membrane phospholipid remodeling, and metabolism into lipid mediators; thus, these PUFAs generally compete for these biological processes in the brain [4,5]. Consequently, maintaining a proper balance between *n*–6 and *n*–3 PUFAs has high physiological relevance.

During development, ARA and DHA are deposited in the brain [6], and both PUFAs are essential for proper brain development [[7], [8], [9], [10], [11], [12], [13]]; however, modern agriculture has led to foods that are high in *n*–6 PUFAs and low in *n*–3 PUFAs [14,15]. To investigate the neurodevelopmental outcome due to exposure to a diet high in LA and low in ALA, we fed pregnant mice either a control diet that contained sufficient levels of LA and ALA or an isocaloric diet that contained higher levels of LA and lower levels of ALA than the control diet (we refer to this diet as LA^high^/ALA^low^). We previously found that offspring exposed in utero to the LA^high^/ALA^low^ diet had fewer glutamatergic neurons in the neocortex [5] and more dopaminergic neurons in the midbrain [16] compared with offspring born to mothers fed the control diet. Furthermore, we previously found that maternal consumption of the LA^high^/ALA^low^ diet either during gestation alone or during gestation and early lactation led to higher sucrose intake [16] and anxiety-related behaviors [5,17] in adult offspring, respectively, compared with offspring born to mothers fed the control diet. Importantly, the offspring in both maternal diet groups were raised postnatally on a diet with sufficient levels of LA and ALA. Thus, these studies indicate that the balance between *n*–6 and *n*–3 PUFAs in the mother’s diet during pregnancy can have a significant effect not only on the offspring’s developing brain but also on their subsequent behaviors. However, we do not yet fully understand the behavioral abnormalities that occur in offspring born to mothers fed the LA^high^/ALA^low^ diet.

A comprehensive behavioral test battery is a combination of several different types of behavioral tests and is frequently used to investigate effects of various experimental manipulations such as genetic modification or drug administration on animal behaviors [18]. Although there are some debates as to which behavioral tests should be combined, the behavioral test battery has been refined to include tests that assess higher brain functions such as emotion and learning [19,20]. Here, we utilized our comprehensive behavioral test battery [[21], [22], [23]] for analyses of both male and female mice that were exposed in utero to either the control diet or the LA^high^/ALA^low^ diet, in order to obtain unbiased and systematic data on their behavioral properties.

## Methods

### Animals

C57BL/6J mice were obtained from Clea Japan and housed at Hiroshima University or University of Toyama under a standard 12-h light/12-h dark schedule. Food and water were available ad libitum. All animal experiments were performed in accordance with the NIH Guidelines for the Care and Use of Laboratory Animals and were approved by the committee for animal experimentation of our universities (Hiroshima University, Permission No. A20–34, A22–51, and A23–51; University of Toyama, Permission No. A2019OPR-2 and A2022OPR-2).

### Diets

The experimental outline was shown in Supplemental Figure 1. Virgin female mice 11 wk of age were fed either the control diet or the LA^high^/ALA^low^ diet (Table 1), both of which were manufactured by Clea Japan. The control diet and the LA^high^/ALA^low^ diet contained canola oil and high-linoleic safflower oil, respectively, as previously reported [5,8,17]. Consequently, the control diet contained sufficient amounts of LA and ALA for rodent development [24,25], and the LA^high^/ALA^low^ diet contained more LA and less ALA compared with the control diet (Table 2). The LA^high^/ALA^low^ diet can be regarded as the *n*–3 PUFA-deficient diet [8]. After 2 wk of consuming either diet, the female mice were mated with male mice that were fed a standard laboratory feed pellets (the CE-2 diet, Clea Japan) and then continued to receive their respective diet through gestation. On the day of birth, the mothers were fed the CE-2 diet in place of the control diet or the LA^high^/ALA^low^ diet; this standard diet contained 44.4% LA, 3.3% ALA, 2.3% eicosapentaenoic acid, and 1.3% DHA [26]. We did not normalize the number of offspring at birth, because there was no statistically significant difference in the litter sizes between the control and LA^high^/ALA^low^ groups [27]. At 3 wk of age, the offspring were weaned, group-housed at 2–5 mice per cage, and continued to receive the CE-2 diet. Both male and female offspring from ≥3 separate litters were used for the experiments.

**TABLE 1:** Composition of the diets.

Ingredient	Amount (g/100 g diet)	
	Control diet	LA^high^/ALA^low^ diet
Corn starch	47	47
Maltodextrin	6.0	6.0
Sucrose	10	10
Canola oil	7.0	0
High-linoleic safflower oil	0	7.0
t-butylhydroquinone	0.0014	0.0014
Milk casein	20	20
L-cystine	0.30	0.30
Cellulose powder	5.0	5.0
AIN-93G mineral mix	3.5	3.5
AIN-93 vitamin mix	1.0	1.0
Choline bitartrate	0.25	0.25
Gardenia pigment	0.025	0.025

Abbreviations: ALA, α-linolenic acid; LA, linoleic acid.

**TABLE 2:** Fatty acid composition of the diets. Fatty acid composition is shown as % of total fatty acids (*n* = 3 diets).

Fatty acid	Control diet	LA^high^/ALA^low^ diet
14:0	0.1% ± 0.0%	0.2% ± 0.0%
16:0	5.2% ± 0.0%	7.9% ± 0.0%
18:0	1.9% ± 0.0%	2.8% ± 0.0%
20:0	0.6% ± 0.0%	0.4% ± 0.0%
22:0	0.3% ± 0.0%	0.3% ± 0.0%
24:0	0.2% ± 0.0%	0.2% ± 0.0%
16:1	0.2% ± 0.0%	0.1% ± 0.0%
18:1	60.2% ± 0.0%	15.3% ± 0.0%
20:1	1.2% ± 0.0%	0.2% ± 0.0%
24:1	0.2% ± 0.0%	0.1% ± 0.0%
18:2*n*–6	19.9% ± 0.0%	71.6% ± 0.1%
18:3*n*–3	9.4% ± 0.0%	0.8% ± 0.0%
Others	0.6% ± 0.0%	0.2% ± 0.1%
Total SFAs	8.3% ± 0.1%	11.7% ± 0.1%
Total MUFAs	61.8% ± 0.0%	15.7% ± 0.0%
Total PUFAs	29.3% ± 0.1%	72.4% ± 0.1%
Total *n*–6 PUFAs	19.9% ± 0.0%	71.6% ± 0.1%
Total *n*–3 PUFAs	9.4% ± 0.0%	0.8% ± 0.0%
*n*–6/*n*–3	2.1 ± 0.0	86.2 ± 3.4

Abbreviations: ALA, α-linolenic acid; LA, linoleic acid.

The control and LA^high^/ALA^low^ diets were stored in the dark at 4°C with oxygen absorbers and were prepared fresh every 3 mo or less. The diets in the cages were replaced completely with fresh diets twice a week. We previously confirmed that the dietary lipids do not undergo peroxidization under these storage and feeding conditions [16].

### Evaluation of dietary lipids

The fatty acid composition of the diets was measured by Japan Food Research Laboratories using gas chromatography. Briefly, total lipids were extracted from 0.5 g of each diet using the standard acid hydrolysis method and were incubated in a boron trifluoride methanol complex methanol solution at 100°C for 9 min to induce transmethylation of fatty acid residues. Fatty acid methyl esters were then extracted using hexane, and 1.0 mL of extract was analyzed using gas chromatography. Gas chromatograph equipped with a flame ionization detector (GC-FID) used was the Agilent 7890B (Agilent Technologies) with the Agilent DB-23 column (Agilent Technologies).

### Evaluation of brain, serum, and liver lipids

The composition of fatty acids in the embryonic brain at embryonic day 14.5 (E14.5), the male adult brain at 10–15 wk of age, the female adult brain at 10–11 wk of age, and maternal serum and liver was measured using gas chromatography. Briefly, 3 embryonic brains that were combined into 1 sample, half of the adult brain, and maternal liver were homogenized with phosphate-buffered saline (PBS). Maternal serum and the homogenates were incubated in methanol solution containing acetyl chloride at 100°C for 60 min to induce transmethylation of fatty acid residues. Fatty acid methyl esters were then extracted using octane, and 1.0 mL of extract was analyzed using gas chromatography. GC-FID used was the GC-2014 (Shimadzu) with the Agilent DB-WAX column (Agilent Technologies).

### Behavioral analyses

Both male and female offspring were subjected to a battery of behavioral tests. Unless stated otherwise, each test was performed between 8:30 am and 6:00 pm. Several offspring were excluded from the analyses (see Supplemental Table 1). The behavioral tests were performed in the sequence below, with an interval of ≥1 d between tests. For details, see Supplemental Methods. Information regarding each offspring and the behavioral data collected in this study are available in the “Mouse Phenotype Database” (http://www.mouse-phenotype.org/).

To evaluate communication-related behavior, we measured maternal separation–induced ultrasonic vocalization (USV) in the offspring on postnatal day 7. To screen for neurological deficits, the righting reflex, whisker twitch reflex, ear twitch reflex, body weight, and rectal temperature were measured in the offspring at 11–12 wk of age. To measure neuromuscular strength, the grip strength test and the wire hang test were performed in the offspring at 11–12 wk of age, as previously described [23]. To measure locomotor activity and anxiety-related behaviors, the light/dark transition test, the open field test, and the elevated plus maze test were performed in the offspring at 11–12 wk of age (for the light/dark transition and open field tests) or in male and female offspring at 11–12 and 12–13 wk of age, respectively (for the elevated plus maze test), as previously described [23,28]. One male control offspring was excluded from the analyses of the elevated plus maze test because it dropped from the open arm. To evaluate sensitivity to painful stimuli, we performed the hot plate test on the offspring at 12–13 wk of age, as previously described [23]. To evaluate social behaviors, we performed the social interaction test in the offspring at 12–13 wk of age, as previously described [23]. To measure motor coordination and balance, we performed the rotarod test in male and female offspring at 12–13 and 13–14 wk of age, respectively, as previously described [23]. As an additional measure of social behaviors, we performed Crawley’s sociability and social novelty preference test in the offspring at 13–14 wk of age, as previously described [23]. To measure sensorimotor gating, the startle response and prepulse inhibition test was performed in male and female offspring at 14–15 and 15–16 wk of age, respectively, as previously described [23]. To measure depression-related behaviors, we performed the Porsolt forced swim test in the offspring at 15–16 wk of age, as previously described [23]. To measure pattern separation, we performed the place recognition test in the offspring at 17–18 wk of age, as previously described [29]. To measure spontaneous recognition memory, we performed the object location test in male and female offspring at 18–19 and 19–20 wk of age, respectively, as previously described [23]. To measure spatial working memory, we performed the spontaneous alternation task in male and female offspring at 19–20 and 20–21 wk of age, respectively, as previously described [29]. To measure spatial reference memory, we performed the Barnes maze test in male and female offspring at 24–31 and 26–34 wk of age, respectively, as previously described [30]. To measure home cage activity, we monitored locomotor activity in the home cage of male and female offspring at 32–34 and 41–44 wk of age, respectively, as previously described [31]. One male control offspring was excluded from the analyses of data obtained in the second week because it died before the test. To measure voluntary running activity in the home cage, male and female offspring at 34–37 and 43–46 wk of age, respectively, were given free access to a single running wheel placed in the home cage after measuring home cage activity. Two male control offspring were excluded from the analyses of data obtained in the first and second weeks because they died before the test. Five female control offspring and 4 female LA^high^/ALA^low^ offspring were excluded from the analyses of data obtained in the first week due to technical problems with the video-analysis system. Two male LA^high^/ALA^low^ offspring were excluded from the analyses of data obtained in the second week due to a machine trouble. To measure fear memory, we performed the contextual and cued fear conditioning test in the offspring at 55–60 wk of age, as previously described [32]. Two male and one female control offspring were excluded from the analyses because they died before the test. To measure social behaviors in an acclimated environment, we performed the social interaction test in the home cage of male and female offspring at 60–62 and 61–63 wk of age, respectively, as previously described [31]. Two male and one female control offspring were excluded from the analyses because they died before the test.

Behavioral data were obtained automatically using ImageLD [28], ImageEP [33], ImageSI [34], ImagePS [35], ImageOF [29], ImageTM [29], ImageBM [30], ImageHA [31], and ImageFZ [32] based on the publicly available NIH Image and ImageJ programs, modified for each test. These plugins are freely available via the “Mouse Phenotype Database” website (http://www.mouse-phenotype.org/software.html) [36].

### Statistical analysis

Statistical analyses were performed using SPSS Statistics version 28 (IBM). Data were analyzed using an unpaired Student’s *t*-test, a Welch’s *t*-test, a Mann‒Whitney’s *U* test, a 2-way or 3-way analysis of variance (ANOVA), or a 2-way or 3-way repeated measures ANOVA. Post hoc simple main effect analysis was performed to compare each dietary group, when necessary. Normality and homoscedasticity of the data were analyzed using a Shapiro‒Wilk’s test and an *F*-test, respectively. Sphericity of the data was analyzed using a Mauchly’s sphericity test, and the data were adjusted using Greenhouse–Geisser, when necessary. Differences with a *P* value of <0.05 were considered statistically significant. We indicated a *P* value as numbers (for ANOVAs) or asterisks (for simple main effect analysis) in each panel of the figure when each difference was statistically significant. All summary data in the figure are expressed as the mean ± SEM. All statistical data are summarized in Supplemental Table 2.

## Results

### Consuming the LA^high^/ALA^low^ diet during pregnancy induces higher *n*–6 PUFAs and lower *n*–3 PUFAs in the embryonic brain and in maternal serum and liver

We first examined the effect of maternal consumption of the LA^high^/ALA^low^ diet on the fatty acid profile in the offspring’s brain by measuring the fatty acid composition in the offspring’s brain at E14.5 and at 10–15 wk of age. We found that *n*–6 and *n*–3 PUFAs were significantly higher and lower, respectively, in the LA^high^/ALA^low^ embryonic brain than age-matched controls (Table 3). In contrast, we measured a slight but significant decrease in 18:1*n*–7 and the *n*–6/*n*–3 ratio, as well as a slight but significant increase in 22:4*n*–6 in the brain of adult LA^high^/ALA^low^ offspring compared with age-matched controls (Supplemental Table 3). These data suggest that most of the changes in the fatty acid profile observed in the embryonic brain due to maternal consumption of the LA^high^/ALA^low^ diet are no longer present after postnatal consumption of the standard diet, consistent with previous studies [17,37]. We also confirmed that *n*–6 and *n*–3 PUFAs were significantly higher and lower, respectively, in serum and liver of mother mice fed the LA^high^/ALA^low^ diet than those of mice fed the control diet when their embryos were at E14.5 (Supplemental Tables 4 and 5). Thus, in utero exposure to the LA^high^/ALA^low^ diet causes a clear—but transient—increase in *n*–6 PUFAs and decrease in *n*–3 PUFAs in the embryonic brain.

**TABLE 3:** Brain fatty acid profiles measured in offspring at E14.5 exposed in utero to the control or LA^high^/ALA^low^ diet.

Fatty acid	Control male offspring	LA^high^/ALA^low^ male offspring	Control female offspring	LA^high^/ALA^low^ female offspring
14:0	2.9% ± 0.1%	3.0% ± 0.1%	2.9% ± 0.1%	3.0% ± 0.1%
16:0	34.4% ± 0.4%	34.8% ± 0.4%	34.3% ± 0.4%	35.0% ± 0.1%
18:0	17.3% ± 0.2%	17.5% ± 0.3%	17.1% ± 0.2%	17.6% ± 0.3%
20:0^1^^,^^2^	0.2% ± 0.0%^3^	0.0% ± 0.0%^3^	0.0% ± 0.0%	0.1% ± 0.0%
16:1	2.2% ± 0.2%	2.4% ± 0.1%	2.0% ± 0.0%	2.2% ± 0.2%
18:1*n*–7	3.2% ± 0.1%	3.2% ± 0.2%	3.2% ± 0.2%	3.1% ± 0.2%
18:1*n*–9	15.4% ± 0.2%	14.2% ± 1.1%	16.4% ± 0.1%	15.6% ± 0.5%
24:1^2^	0.1% ± 0.0%^3^	0.0% ± 0.0%^3^	0.1% ± 0.0%	0.1% ± 0.0%
18:2*n*–6^1^	0.6% ± 0.1%	1.0% ± 0.1%	0.6% ± 0.1%	1.1% ± 0.1%
20:4*n*–6^1^	8.8% ± 0.2%	10.9% ± 0.5%	9.6% ± 0.3%	11.2% ± 0.3%
22:4*n*–6^1^	1.9% ± 0.0%	2.7% ± 0.1%	2.0% ± 0.1%	2.8% ± 0.1%
22:5*n*–6^1^^,^^2^	0.2% ± 0.0%^3^	3.9% ± 0.3%^3^	0.2% ± 0.0%^4^	3.1% ± 0.2%^4^
22:6*n*–3^1^	11.8% ± 0.4%	5.6% ± 0.7%	10.6% ± 0.3%	4.5% ± 0.2%
Total SFAs	54.7% ± 0.7%	55.5% ± 0.5%	54.4% ± 0.7%	55.8% ± 0.3%
Total MUFAs	21.4% ± 0.0%	20.2% ± 1.1%	22.1% ± 0.2%	21.3% ± 0.5%
Total *n*–6 PUFAs^1^	11.6% ± 0.2%	18.7% ± 0.7%	12.7% ± 0.6%	18.3% ± 0.5%
Total *n*–3 PUFAs^1^	12.2% ± 0.4%	5.6% ± 0.7%	10.8% ± 0.3%	4.6% ± 0.2%
*n*–6/*n*–3^1^	1.0 ± 0.0	3.4 ± 0.2	1.2 ± 0.0	4.0 ± 0.1

Abbreviations: ALA, α-linolenic acid; LA, linoleic acid.

*n* = 3/group. Data were analyzed using a 2-way ANOVA. Fatty acids that contribute to >1% of total fatty acids in either the embryonic or adult brain are shown (see also Supplemental Table 3).

1 Diet effect is significant (*P* < 0.05, 2-way ANOVA).

2 Diet x sex interaction is significant (*P* < 0.05, 2-way ANOVA).

3 There is a significant difference between the control and LA^high^/ALA^low^ male offspring (*P* < 0.05, simple main effect analysis).

4 There is a significant difference between the control and LA^high^/ALA^low^ female offspring (*P* < 0.05, simple main effect analysis).

### Consuming the LA^high^/ALA^low^ diet during pregnancy does not affect body weight or food intake of mothers or offspring

We next examined the consequences of consuming the LA^high^/ALA^low^ diet in the mother and offspring. First, we found that the change in body weight was similar between mothers fed the LA^high^/ALA^low^ diet and mothers fed the control diet (Supplemental Figure 2A), as well as between their offspring (Supplemental Figure 2B). Moreover, daily food intake was similar between the 2 dietary groups for both the mothers (Supplemental Figure 2C) and their offspring (Supplemental Figure 2D), consistent with our previous reports [16,17]. These data indicate that maternal consumption of the LA^high^/ALA^low^ diet during pregnancy does not affect the mother or offspring’s weight gain or food intake.

### In utero exposure to the LA^high^/ALA^low^ diet induces hyperactivity in adult female offspring and lower voluntary wheel running in male offspring

**FIGURE 1:**
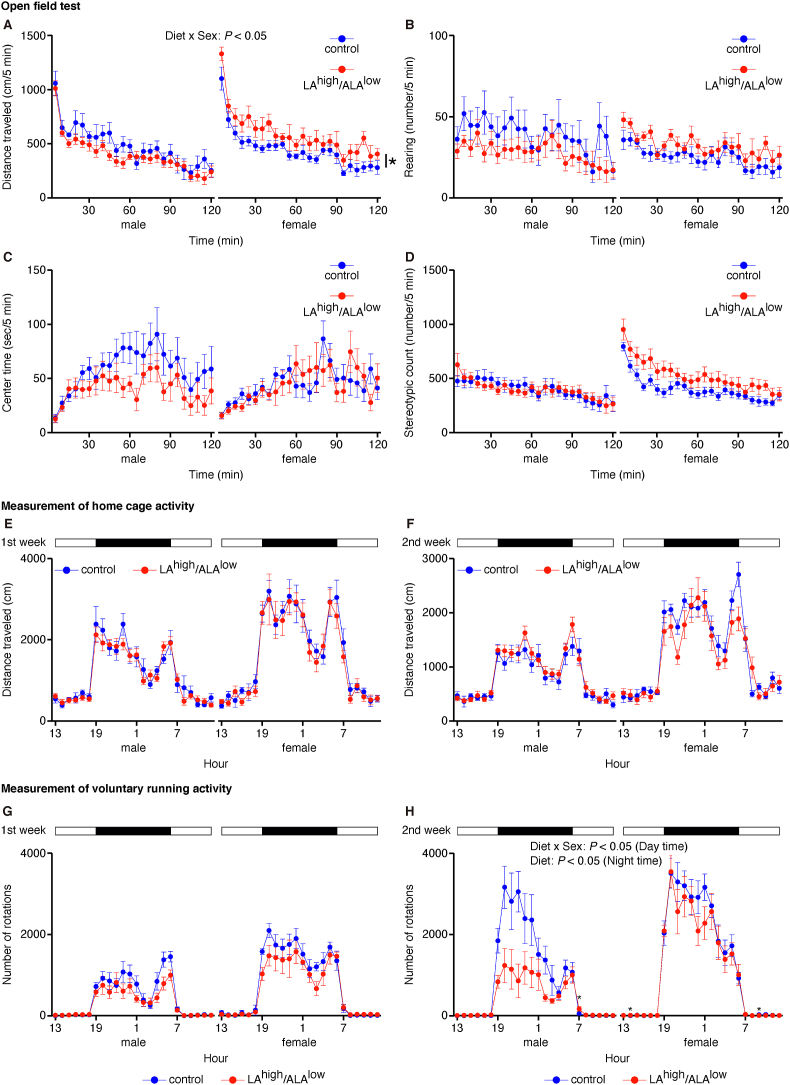
Locomotor activity, anxiety-related behaviors, home cage activity, and voluntary running measured in offspring exposed in utero to the control or LA^high^/ALA^low^ diet. (A–D**)** Time course of distance traveled (**A**), frequency of rearing events (B), time spent in the center zone (C), and frequency of stereotypic events (D) in the open field test measured in adult offspring (*n* = 8–11/group). Data were analyzed using a 3-way ANOVA (with time as a repeated measure) with post hoc simple main effect analysis for multiple comparisons. (E and F) Distance traveled during the first week (E) and second week (F) in the home cage activity test measured in adult offspring (*n* = 7–11/group). (G and H**)** Number of rotations of the running wheel during the first week (G) and second week (H) in the wheel running test measured in adult offspring (*n* = 6–11/group). In (E-H), the light and dark periods are indicated by white and black bars, respectively, and data were analyzed using a 3-way ANOVA (with hour as a repeated measure) with post hoc simple main effect analysis for multiple comparisons. ∗*P* < 0.05 (simple main effect analysis). ALA, α-linolenic acid; ANOVA, analysis of variance; LA, linoleic acid.

Next, we assessed whether in utero exposure to the LA^high^/ALA^low^ diet has long-term effects on locomotor activity and/or anxiety-related behaviors using the open field test at 11–12 wk of age. This behavioral test was performed using a device that was not familiar to the offspring and, therefore, reflected behaviors observed in a novel environment. Male offspring in the LA^high^/ALA^low^ group traveled a similar distance as control males; in contrast, the female offspring in the LA^high^/ALA^low^ group traveled a slight but significantly longer distance compared with control females (Figure 1A). Moreover, we found no significant difference between LA^high^/ALA^low^ and control offspring with respect to the frequency of rearing (Figure 1B), time spent in the center zone (Figure 1C), or the frequency of stereotypic counts (Figure 1D), regardless of sex. These results suggest that adult mice that were exposed in utero to the LA^high^/ALA^low^ diet do not exhibit higher anxiety-related behaviors, with the exception of a slight but significant increase in activity measured in female offspring in an unacclimated open field.

Next, we examined locomotor activity in an acclimated environment at 32–34 wk of age for male offspring and at 41–44 wk of age for female offspring. We first measured locomotor activity in the home cage for 2 wk and found no difference between control and LA^high^/ALA^low^ male or female offspring (Figure 1E and 1F). We then assessed voluntary locomotor activity using a running wheel placed in the home cage for 2 wk at 34–37 wk of age for male offspring and at 43–46 wk of age for female offspring. We found no difference in the first week (Figure 1G); however, in the second week, wheel-running activity was lower in the LA^high^/ALA^low^ offspring compared with control offspring, particularly during nighttime; moreover, this difference between groups was more pronounced in the male offspring than in the female offspring (Figure 1H). Thus, in utero exposure to the LA^high^/ALA^low^ diet has relatively little effect on spontaneous locomotor activity in the home cage in both male and female offspring, but reduces voluntary wheel running, particularly in male offspring.

### In utero exposure to the LA^high^/ALA^low^ diet impairs social behaviors in adulthood

**FIGURE 2:**
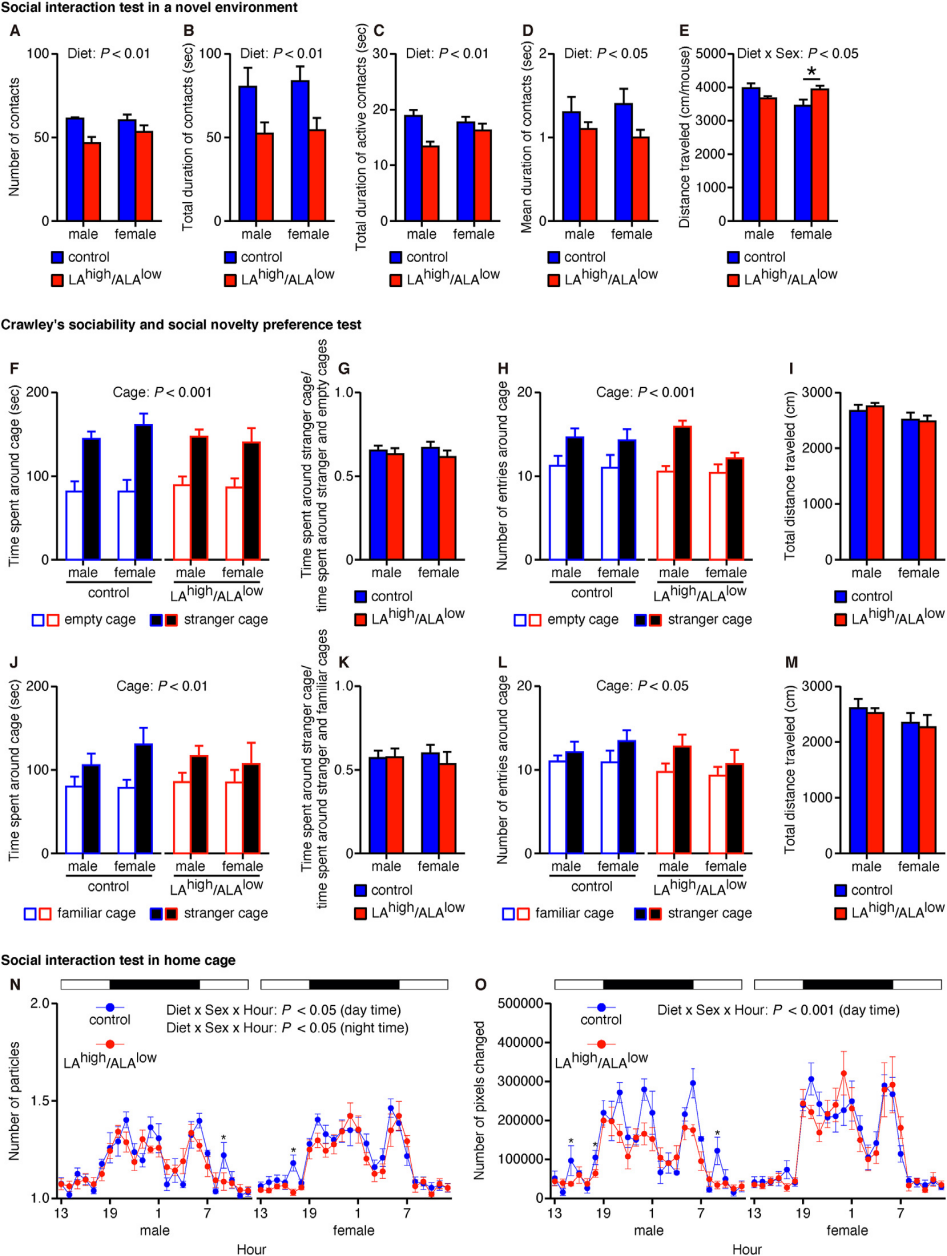
Social behaviors measured in offspring exposed in utero to the control or LA^high^/ALA^low^ diet. (A–E) Summary of the number of contacts (A), the total duration of contacts (B), the total duration of active contacts (C), the mean contact duration (D), and distance traveled (E) measured using the social interaction test in a novel environment (*n* = 4–5/group). Data were analyzed using a 2-way ANOVA with post hoc simple main effect analysis for multiple comparisons. (F–I) Summary of the time spent around each cage (F), the relative time spent around the cage with a stranger mouse (G), the number of entries around each cage (H), and the total distance traveled (I) measured using Crawley’s sociability test (*n* = 8–11/group). Data were analyzed using a 2-way ANOVA (G and I) or a 3-way ANOVA (F and H). (J–M) Summary of the time spent around each cage (J), the relative time spent around the cage with a stranger mouse (K), the number of entries around each cage (L), and the total distance traveled (M) measured using Crawley’s social novelty preference test (*n* = 8–11/group). Data were analyzed using a 2-way ANOVA (K and M) or a 3-way ANOVA (J and L). (N and O) The number of particles (i.e. contacts, N) and the number of pixels changed (i.e. the activity level, O) measured using the social interaction test in the home cage (*n* = 3–5/group). Data were analyzed using a 3-way ANOVA (with hour as a repeated measure). ∗*P* < 0.05 (simple main effect analysis). ALA, α-linolenic acid; ANOVA, analysis of variance; LA, linoleic acid.

Next, we examined social behaviors using the social interaction test in a novel (i.e. unacclimated) environment at 12–13 wk of age. We found that the number of contacts, total duration of contacts, total duration of active contacts, and mean duration of contacts were lower in both male and female LA^high^/ALA^low^ offspring than sex-matched controls (Figure 2A–D). Moreover, we found that female—but not male—LA^high^/ALA^low^ offspring traveled a longer distance compared with sex-matched controls (Figure 2E). Together, these data suggest that in utero exposure to the LA^high^/ALA^low^ diet impairs social behaviors in a novel environment.

To further examine the effect of the LA^high^/ALA^low^ diet on social behaviors, we performed Crawley’s sociability and social novelty preference test using the 3-chambered box at 13–14 wk of age. We found that control and LA^high^/ALA^low^ offspring of both sexes had similar increases in the amount of time spent around the cage containing a stranger mouse compared with time spent around the empty cage (Figure 2F). Moreover, we found no difference between groups with respect to the relative amount of time spent around the cage containing a stranger mouse, regardless of sex (Figure 2G). We also found that both male and female control and LA^high^/ALA^low^ offspring had more entries around the cage containing a stranger mouse compared with the empty cage (Figure 2H). In contrast, we found no significant difference between groups with respect to the total distance, regardless of sex (Figure 2I). Similar results were obtained when we compared the results obtained between the stranger mouse and a familiar mouse (Figure 2J–M). Thus, in utero exposure to the LA^high^/ALA^low^ diet does not appear to affect the offspring’s sociability measured using Crawley’s 3-chambered system.

We also examined social behaviors in the offspring at 60–62 wk of age for male offspring and at 61–63 wk of age for female offspring in an acclimated environment for 1 week. Using this approach, we found that the number of contacts was similar between control and LA^high^/ALA^low^ offspring during most time periods, although a slight but significant decrease was observed in both the male and female LA^high^/ALA^low^ offspring compared with their sex-matched controls at several times during the daytime (Figure 2N). Similarly, locomotor activity was similar between the control and LA^high^/ALA^low^ offspring during most time periods, with a slight but significant decrease observed in the male LA^high^/ALA^low^ offspring compared with sex-matched controls at several times during the daytime (Figure 2O). These results suggest that in utero exposure to the LA^high^/ALA^low^ diet may impair sociability in an acclimated environment in certain time periods.

### In utero exposure to the LA^high^/ALA^low^ enhances recognition memory in adulthood

**FIGURE 3:**
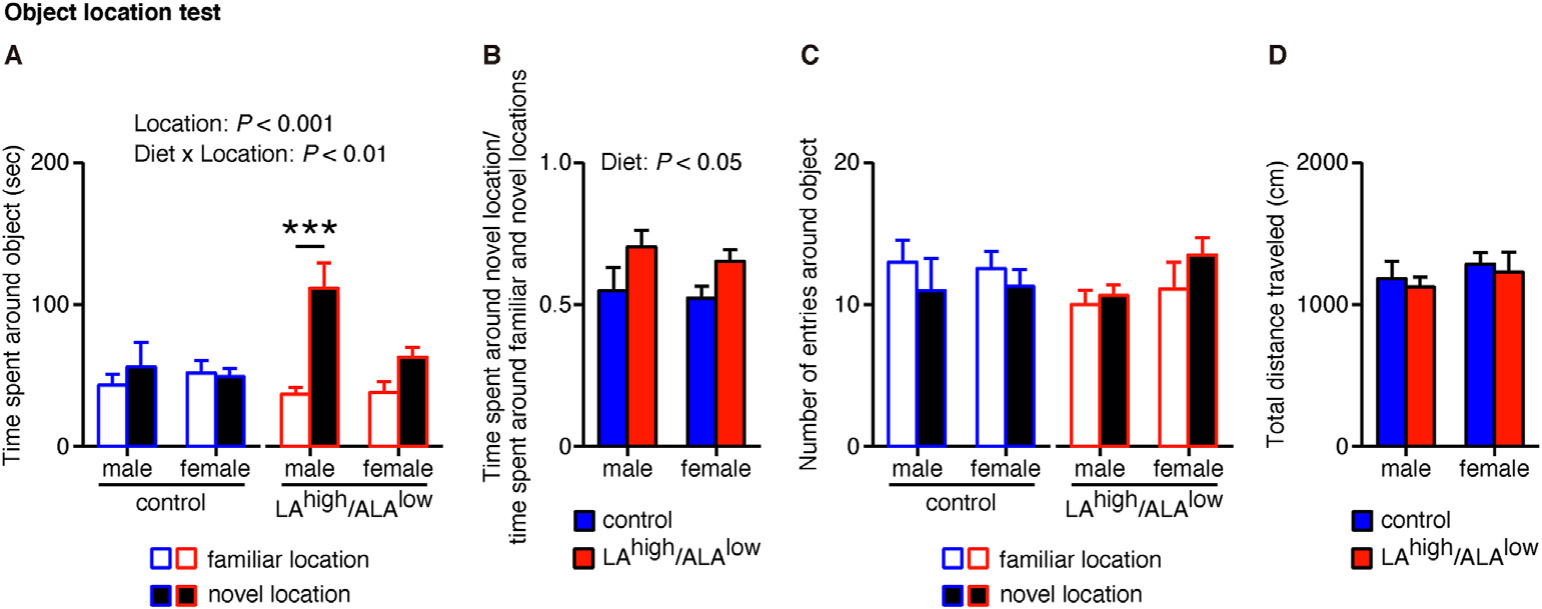
Recognition memory measured in offspring exposed in utero to the control or LA^high^/ALA^low^ diet. (A–D) Summary of the time spent around each object (A), the time spent around the object in a novel location relative to the total time spent around both the novel and familiar locations (B), the number of entries around each object (C), and the total distance traveled (D) measured using the object location test (*n* = 8–11/group). Data were analyzed using a 2-way ANOVA (B and D) or a 3-way ANOVA (A and C) with post hoc simple main effect analysis for multiple comparisons. ∗∗∗*P* < 0.001 (simple main effect analysis). ALA, α-linolenic acid; ANOVA, analysis of variance; LA, linoleic acid.

We next examined spontaneous recognition memory using the object location test at 18–19 wk of age for male offspring and at 19–20 wk of age for female offspring and found that the male LA^high^/ALA^low^ offspring spent significantly more time around the object in a novel location compared with sex-matched controls (Figure 3A). In addition, we found a significant difference between LA^high^/ALA^low^ and control offspring with respect to the ratio between time spent around the object in a novel location and the total time spent around the objects in both the familiar and novel locations (Figure 3B). In contrast, we found no significant difference between groups with respect to the number of entries around the object in either location (Figure 3C), or in the total distance traveled (Figure 3D), regardless of sex. Together, these data suggest that in utero exposure to the LA^high^/ALA^low^ diet improves spontaneous recognition memory measured using the object location test.

### In utero exposure to the LA^high^/ALA^low^ impairs retention of newly learned spatial memory in adult female offspring

**FIGURE 4:**
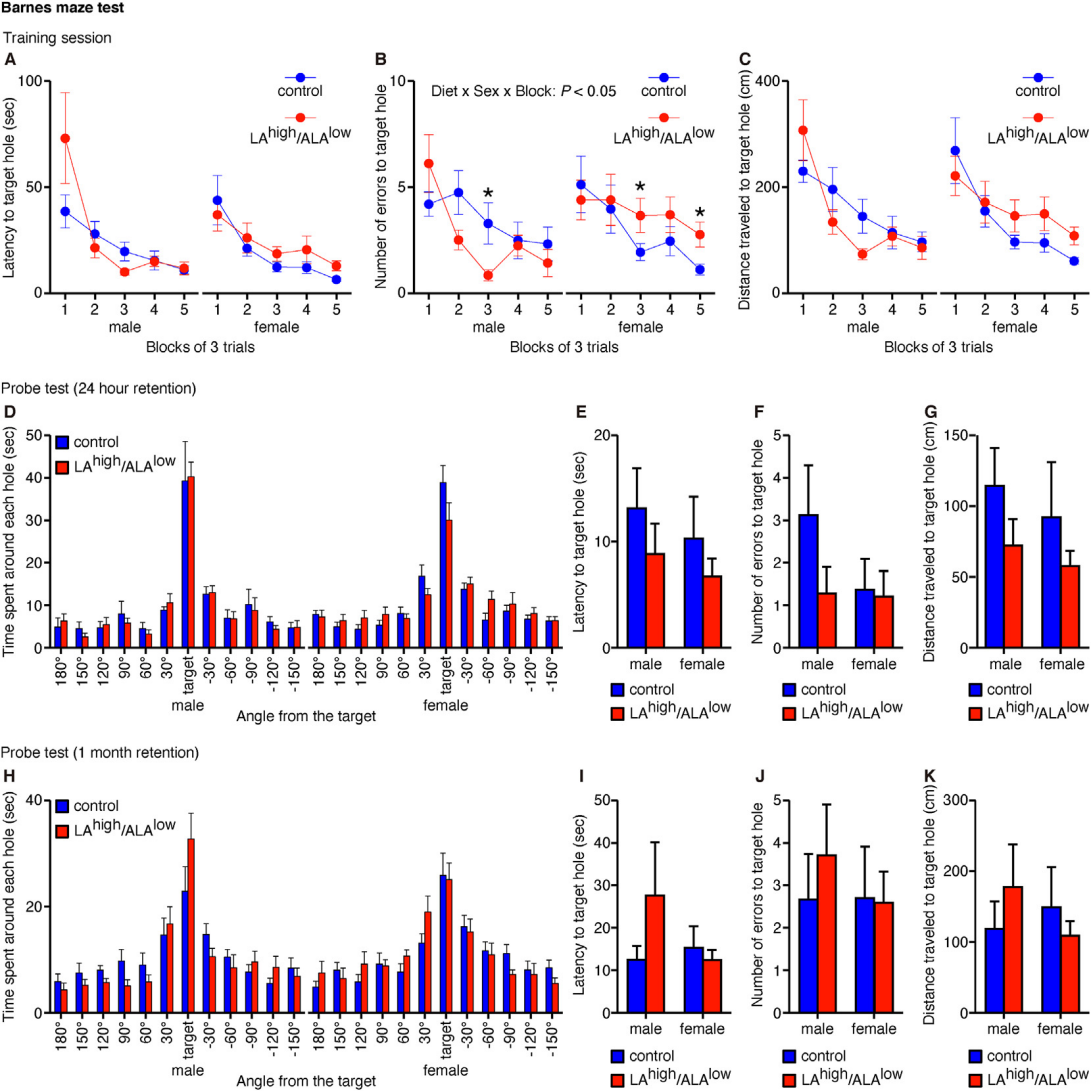
Spatial reference memory measured in offspring exposed in utero to the control or LA^high^/ALA^low^ diet. (A–C) Summary of the latency to reach the target hole (A), the number of errors before reaching the target hole (B), and the distance traveled to reach the target hole (C) during the training session of the Barnes maze test (*n* = 8–11/group). Data were analyzed using a 3-way ANOVA (with block as a repeated measure) with post hoc simple main effect analysis for multiple comparisons. (D–K) Summary of the time spent around each hole (D and H), the latency to reach the target hole (E and I), the number of errors before reaching the target hole (F and J), and the distance traveled to reach the target hole (G and K) in the probe test of the Barnes maze test performed 1 d (D-G) and 1 mo (H-K) after the training session (*n* = 8–11/group). Data were analyzed using a 2-way ANOVA. Data were analyzed using a 2-way ANOVA. ∗*P* < 0.05 (simple main effect analysis). ALA, α-linolenic acid; ANOVA, analysis of variance; LA, linoleic acid.

Next, we examined whether in utero exposure to the LA^high^/ALA^low^ diet affects spatial reference memory using the Barnes maze test at 24–30 wk of age for male offspring and at 26–33 wk of age for female offspring. We found that during the training session, the number of errors before reaching the target hole was lower in the LA^high^/ALA^low^ male offspring but higher in the LA^high^/ALA^low^ female offspring compared with sex-matched controls (Figure 4B), despite no difference between groups with respect to the latency to reach the target hole (Figure 4A) or the distance traveled to reach the target hole (Figure 4C), regardless of sex. We then performed probe trials in order to evaluate spatial memory retention 1 day and 1 mo after the training session, but found no difference between groups with respect to the amount of time spent around the target hole (Figure 4D and H), the latency to reach the target hole (Figure 4E and I), the number of errors before reaching the target hole (Figure 4F and J), or the distance traveled to reach the target hole (Figure 4G and K), regardless of sex. Thus, in utero exposure to the LA^high^/ALA^low^ diet affects spatial learning but not appear to affect spatial memory retention.

**FIGURE 5:**
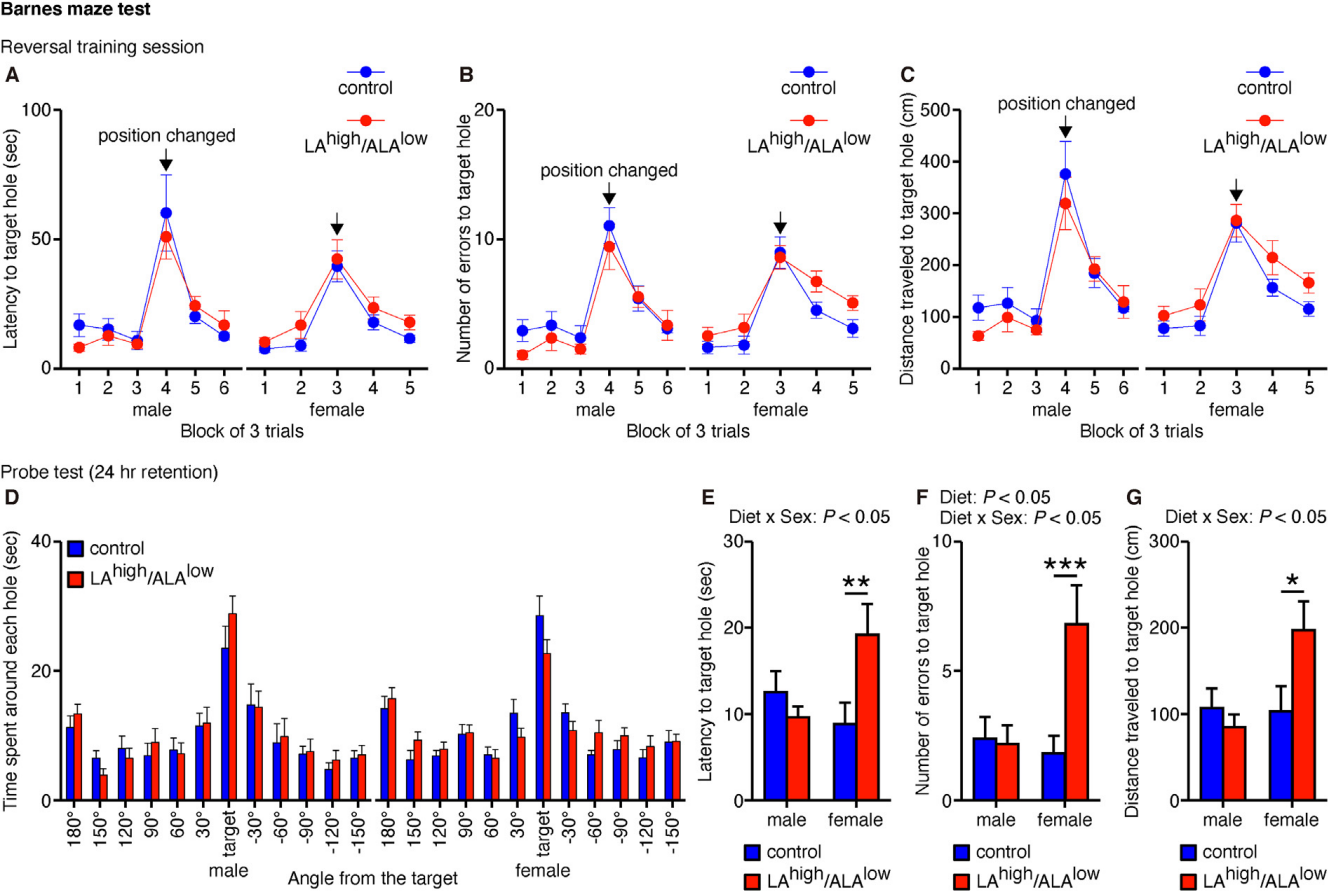
Flexibility of spatial reference memory measured in offspring exposed in utero to the control or LA^high^/ALA^low^ diet. (A–C) Summary of the latency to reach the target hole (**A**), the number of errors before reaching the target hole (B), and the distance traveled to reach the target hole (C) in the training session of the Barnes maze test (*n* = 8–11/group). Data were analyzed using a 2-way ANOVA (with block as a repeated measure). (D–G) Summary of the time spent around each hole (D), the latency to reach the target hole (E), the number of errors before reaching the target hole (F), and the distance traveled to reach the target hole (G) in the probe test of the Barnes maze test performed 1 d after the training session (*n* = 8–11/group). Data were analyzed using a 2-way ANOVA with post hoc simple main effect analysis for multiple comparisons. ∗*P* < 0.05, ∗∗*P* < 0.01, and ∗∗∗*P* < 0.001 (simple main effect analysis). ALA, α-linolenic acid; ANOVA, analysis of variance; LA, linoleic acid.

We then examined the flexibility of spatial reference memory by performing the reversal learning task of the Barnes maze test at 30–31 wk of age for male offspring and at 33–34 wk of age for female offspring, in which the location of the target hole was repositioned 180° during the training session. We found no difference between groups with respect to the latency to reach the target hole (Figure 5A), the number of errors before reaching the target hole (Figure 5B), or the distance traveled to reach the target hole (Figure 5C)—regardless of sex—before or after changing the location of the target hole. However, in the probe trials performed 1 day after the training session, the female LA^high^/ALA^low^ offspring—but not the male LA^high^/ALA^low^ offspring—had a higher latency to reach the target hole (Figure 5E), made more errors before reaching the target hole (Figure 5F), and traveled a greater distance before reaching the target hole (Figure 5G) compared with sex-matched controls, despite no difference in the time spent around the target hole (Figure 5D). Together, these data suggest that in utero exposure to the LA^high^/ALA^low^ diet impairs the retention of newly learned spatial memory.

### In utero exposure to the LA^high^/ALA^low^ affects wire hang performance in adult female offspring

We also investigated USVs (Supplemental Figure 3), physical characteristics and motor coordination (Supplemental Figure 4), anxiety-related behaviors (Supplemental Figure 5), sensorimotor gating (Supplemental Figure 6), depressive behavior (Supplemental Figure 7), pattern separation (Supplemental Figure 8), spatial working memory (Supplemental Figure 9), and fear conditioning (Supplemental Figures 10 and 11), and we found that wire hang performance in the wire hang test was affected in the female LA^high^/ALA^low^ offspring (see Supplemental Results).

## Discussion

Given that both *n*–6 and *n*–3 PUFAs are required for fetal brain development and are provided solely from the mother’s diet, current global changes in our dietary *n*–6 and *n*–3 PUFAs warrant a systematic understanding of the effect of a dietary imbalance between *n*–6 and *n*–3 PUFAs during pregnancy on the child’s subsequent development and behaviors. In many nutritional studies, researchers fed the animals a diet high in *n*–6 PUFAs and/or low in *n*–3 PUFAs throughout gestation, lactation, and postweaning, often continuing this diet for several generations, and then investigated the effects on specific behaviors (for review, see [[38], [39], [40]]). This long-term dietary imbalance between *n*–6 and *n*–3 PUFAs was shown to induce a significant increase in *n*–6 PUFAs and a decrease in *n*–3 PUFAs in the adult brain, which can manifest in certain behavioral deficits [41]. Here, we fed pregnant mice the LA^high^/ALA^low^ diet only during gestation—switching to the standard diet after birth—in order to exclude any postnatal dietary effects on the offspring’s behaviors; we then performed a comprehensive behavioral test battery on the offspring in order to systematically catalog the effects of in utero exposure to the LA^high^/ALA^low^ diet on various behaviors. Interestingly, we found that in utero exposure to the LA^high^/ALA^low^ diet decreased social behaviors and improved recognition memory in both male and female mice. We also found that male LA^high^/ALA^low^ offspring have reduced voluntary running and improved spatial learning, whereas female LA^high^/ALA^low^ offspring also have hyperactivity, impaired spatial learning, and an impaired flexibility of spatial memory retention. Thus, our results may provide a fundamental roadmap for the relationship between maternal PUFA intake and long-term behavioral changes in the offspring.

How might a maternal dietary imbalance between *n*–6 and *n*–3 PUFAs affect the offspring’s behavioral properties? One possible mechanism is that *n*–6 and *n*–3 PUFAs in the brain may modulate neuronal synaptic plasticity; for example, lifelong exposure to an African peanut oil—which is high in LA and low in ALA—from gestation to adulthood was shown to increase *n*–6 PUFAs and decrease *n*–3 PUFAs in the mouse brain, impairing endocannabinoid system–mediated long-term depression of neurons in the prefrontal cortex, resulting in increased anxiety-related behaviors and depressive behaviors [42]. Here, we show that although in utero exposure to the LA^high^/ALA^low^ diet induced higher *n*–6 PUFAs and lower *n*–3 PUFAs in the embryonic brain, these changes did not carry through to adulthood; this recovery of brain PUFA levels by consuming a postnatal diet sufficient in *n*–3 PUFAs has been reported previously [17,37]. Thus, a transient increase in *n*–6 PUFAs and/or decrease in *n*–3 PUFAs in the developing brain, which is no longer present in the adult brain, would cause neurodevelopmental abnormalities, which would be expected to trigger behavioral abnormalities in adulthood. In utero exposure to a LA^high^/ALA^low^ diet was previously shown to impair brain development in both the neocortex and midbrain [5,16] via lipid mediators derived from ARA and DHA in cellular membranes [5]. Further studies are necessary to identify what kinds of neurodevelopmental abnormalities contribute to the relationship between a maternal dietary imbalance between *n*–6 and *n*–3 PUFAs and subsequent changes in the offspring’s behavioral properties.

Impaired social behavior is a core symptom of autism spectrum disorder, and environmental factors that cause deficits in social behavior have attracted considerable attention. A previous observational study found that reduced consumption of seafood during pregnancy increases the risk of reduced communication skills in children [43]. Moreover, a previous animal study found that male mice exposed long term to a sunflower oil diet high in LA and low in ALA from gestation through adulthood make fewer contacts with an unfamiliar mouse when placed in a novel environment [44]. In support of this finding, we report that social behaviors were lower in both male and female offspring exposed in utero to the LA^high^/ALA^low^ diet when the offspring were placed in a novel environment. However, we found no apparent impairments in social behavior measured using Crawley’s sociability and social novelty preference test. Lower social approach of mice observed in this test is reminiscent of symptoms observed in patients with autism spectrum disorder [45]. Importantly, the offspring were pre-acclimated to the experimental environment of this test. Thus, it is likely that the control and LA^high^/ALA^low^ offspring in the social interaction test in a novel environment were exposed to stronger environmental stress compared with Crawley’s sociability and social novelty preference test. Furthermore, in the former test, the control and LA^high^/ALA^low^ offspring interacted with mice of the same age, whereas in the latter test, they interacted with younger, smaller mice; thus, the environmental stressor may serve to trigger impaired social behavior in LA^high^/ALA^low^ offspring. Consistent with this notion, we did not observe any overt social impairments in the LA^high^/ALA^low^ offspring in the social interaction test performed in the home cage. Nevertheless, further studies are needed in order to investigate the mechanism underlying the putative link between impaired social behavior in mice exposed in utero to the LA^high^/ALA^low^ diet and environmental stress.

With respect to anxiety-related behaviors, the evidence to date has been somewhat contradictory. For example, we previously reported that anxiety-related behaviors in the open field test and in the elevated plus maze test were higher in both male and female offspring exposed to a safflower oil-containing LA^high^/ALA^low^ diet from conception through postnatal day 10 [5,17]. In the present study, we found that anxiety-related behaviors in the open field test, the light/dark transition test, and the elevated plus maze test were similar between control offspring and offspring that were exposed in utero—but not following birth—to a similar LA^high^/ALA^low^ diet. Although these studies differed in several respects, one likely explanation may be the difference in floor brightness between these experiments. Mice are nocturnal and do not favor a light environment, and in our previous studies, the floor was illuminated at 1160 lux, and anxiety-related behaviors were increased in the LA^high^/ALA^low^ offspring [5,17]; in contrast, in the present study, the floor was illuminated at only 100 lux during the open field test, the light–dark transition test, and the elevated plus maze test, and anxiety-related behaviors were similar between LA^high^/ALA^low^ and control offspring. These data raise a hypothesis that offspring exposed to the LA^high^/ALA^low^ diet have an impaired emotional state and/or regulation in a stressful environment. Recently, we have reported that male offspring exposed in utero to the same LA^high^/ALA^low^ diet as used in this study showed higher anxiety-related behaviors in the open field test under a light environment but not under a dim environment than male offspring exposed in utero to the same control diet [27]. Previous reports also support this hypothesis; for example, mice exposed to an *n*–3 PUFA-deficient artificial milk and diet from lactation through adulthood had higher anxiety-related behaviors during the elevated plus maze test under bright and noisy conditions, but not under dim, quiet conditions [39], and mice exposed to an *n*–3 PUFA-deficient diet from gestation through adulthood had higher anxiety-related behaviors in the novelty-suppressed feeding test when the mice were pre-exposed to stress induced by social isolation [46]. Further studies designed to investigate the synergistic effects of both maternal dietary PUFAs and stress on anxiety-related behaviors in the offspring may help reveal the mechanism that underlies the emotional deficits observed in LA^high^/ALA^low^ offspring.

Recognition memory is used to recall the prior occurrence of a given stimulus. Here, we report that spontaneous recognition memory was higher in both male and female LA^high^/ALA^low^ offspring measured using the object location test. Because this test relies on the animal’s intrinsic curiosity regarding novel stimuli, it is also possible that the LA^high^/ALA^low^ offspring are more curious regarding an object located in an unfamiliar position. Importantly, we previously reported that the number of dopaminergic neurons in the midbrain was higher in both male and female LA^high^/ALA^low^ offspring than sex-matched controls [16], and these neurons respond to the presentation of a novel stimulus [47,48]. Further studies are, therefore, warranted in order to determine which brain functions play a role in the increase in recognition memory observed in the LA^high^/ALA^low^ offspring.

Interestingly, our study revealed sex-based differences in the effects of a maternal dietary imbalance between *n*–6 and *n*–3 PUFAs on the offspring’s behaviors, as we found several behavioral abnormalities in the female offspring but not in the male offspring, including longer latency to fall in the wire hang test, higher locomotion in the open field test and in the social interaction test in a novel environment, shorter time spent immobile in the Porsolt forced swim test, a lower number of errors in spatial learning, and an impaired flexibility in spatial memory retention. These behavioral changes may occur independently, but we must also consider that the apparent hyperactive behavior observed in the female LA^high^/ALA^low^ offspring may indirectly cause other behavioral changes. Indeed, it may be reasonable to speculate that hyperactivity in female LA^high^/ALA^low^ offspring resulted in a shorter time they were immobile in the Porsolt forced swim test. Thus, the behavioral data measured in the female offspring in our study should be interpreted with caution.

This study has several possible limitations that warrant discussion. First, both the control and LA^high^/ALA^low^ diets contain only LA and ALA as the *n*–6 and *n*–3 PUFAs, respectively, although typical human diets contain a variety of *n*–6 and *n*–3 PUFAs. Given that the conversion of LA and ALA to ARA and DHA, respectively, is impaired in humans compared with that in rodents [[49], [50], [51]], functional differences between LA and ARA and between ALA and DHA in a diet should be considered in future animal studies. Second, the *n*–6/*n*–3 ratio in the LA^high^/ALA^low^ diet (86.2 ± 3.4) is considerably higher than many modern human diets, in which the *n*–6/*n*–3 ratio typically ranges from 4 to 20 [15]. In our study, we used a diet with a relatively high *n*–6/*n*–3 ratio due to the mouse’s short gestational period—∼19 d—compared with humans, thus allowing us to rapidly increase and decrease *n*–6 and *n*–3 PUFA levels, respectively. Indeed, this diet rapidly led to higher *n*–6 PUFA and lower *n*-3 PUFA levels in the embryonic mouse brain. We also confirmed that the brain *n*–6/*n*–3 ratio in the adult mouse fed a similar LA^high^/ALA^low^ diet from embryonic periods to adulthood was 1.8 in average, which is within a range of the *n*–6/*n*–3 ratios observed in the postmortem brains of Americans [16]. Thus, the *n*–6/*n*–3 ratio in the brain of our animal model produced by consuming our LA^high^/ALA^low^ diets is, at least partially, compatible with the human brain state. Nevertheless, studies involving animals with a longer gestational period may better reveal the effects of in utero exposure to a diet with an *n*–6/*n*–3 ratio that is closer to a typical Western diet. Third, we fed the pregnant mice the control and LA^high^/ALA^low^ diets only during gestation, changing to a standard diet at birth; however, several differences in neurodevelopmental events have been reported in utero between humans and mice. For example, some neurodevelopmental events that occur postnatally in mice occur during gestational weeks 36–40 in humans [52]. Therefore, these differences between mice and humans should be considered when attempting to extrapolate the effects of a maternal LA^high^/ALA^low^ diet on mouse behaviors to the effects on brain function in a human child.

Many studies have reported that a maternal dietary imbalance between *n*–6 and *n*–3 PUFAs can affect several brain functions in children. For example, increase in the *n*–6/*n*–3 ratio in the maternal diet is inversely associated with the child’s cognitive and psychomotor development [[53], [54], [55]], although maternal intake of either *n*–6 or *n*–3 PUFAs alone is either positively associated [54] or not associated [53] with the child’s neurodevelopment. Studies have also shown that an increase in the mother’s plasma *n*–3/*n*–6 ratio is associated with fewer emotional problems in the child, whereas an increase in maternal plasma DHA or ARA levels alone is associated with fewer emotional problems and more behavioral problems, respectively, in children [56]. Our animal study and the aforementioned human studies provide new insights showing that changes in the maternal dietary balance between *n*–6 and *n*–3 PUFAs can have significant effects in the child’s brain function later in life. Future studies should, therefore, examine the putative association between changes in the *n*–6/*n*–3 ratio in the maternal diet and a variety of behaviors in the child. With respect to animal experiments, future studies are needed in order to elucidate the neurodevelopmental mechanism that induces the various behavioral abnormalities observed in mice exposed in utero to the LA^high^/ALA^low^ diet. Studies using a diet that contains various *n*–6 and *n*–3 PUFAs and lower *n*–6/*n*–3 ratio are also necessary to enhance translational relevance of behavioral data to human situations.

## Author contributions

The authors’ responsibilities were as follows – NS: designed research, wrote the paper, and had primary responsibility for final content; NS, KF, MK, MA, YK, KT, MS: conducted research and analyzed data; and all authors: read and approved the final manuscript.

## Date availability

Data described in the manuscript, code book, and analytic code will be made publicly and freely available without restriction at http://www.mouse-phenotype.org/.

## Funding

This research was supported by JSPS KAKENHI (grant numbers JP16H06276, JP19H05023, JP21H03357, and JP22H04922 to NS) and the Lotte Research Promotion Grant (to NS). This research was also partially supported by the Lotte Research Promotion Grant from the Lotte Foundation, which was not involved in the design, implementation, analysis, and interpretation of the data.

## Conflict of interest

The authors report no conflicts of interest.
