# Supplementary material for: Mice Born to Mothers Fed a Diet High in Omega-6 Fatty Acids and Low in Omega-3 Fatty Acids During Pregnancy Exhibit Various Behavioral Changes Including Impaired Social Behaviors and Enhanced Recognition Memory
Source: J Nutr. 2025 Jan 3;155(3):775–87. doi: 10.1016/j.tjnut.2024.12.031 (PMC11934248; doi:10.1016/j.tjnut.2024.12.031)
Supplement: multimedia component 1 [file mmc1.pdf]

Supplemental data

Mice born to mothers fed a diet high in omega-6 fatty acids and low in omega-3 fatty acids during pregnancy exhibit various behavioral changes including impaired social behaviors and enhanced recognition memory

Sakayori et al.

SUPPLEMENTAL METHODS

Behavioral analyses

Measurement of ultrasonic vocalization

Each offspring was isolated from its mother and placed in an empty glass beaker (diameter 78 mm × height 103 mm), and a microphone was placed 110 mm from the bottom of the glass beaker so that the microphone tip and the center of the beaker were aligned. After recording for 3 minutes, the offspring was returned to its home cage. USV was recorded using an ultrasonic vocalization analysis system (O'Hara & Co., Tokyo, Japan), and the recorded data were analyzed using USVSEG software [1]. The number of vocalizations, the latency to first vocalizations (in sec), the mean duration of each offspring's vocalization (in msec), and mean maximum vocalization frequency (in kHz) were used for statistical analysis.

Neurological screening

In the grip strength test, a grip strength meter (O'Hara & Co.) was used to measure forelimb grip strength, which is expressed in Newtons (N). In the wire hang test, the offspring was placed on a wire mesh, which was then inverted slowly while the offspring gripped the wire in order to hang onto the mesh; the latency to fall from the wire mesh was recorded, with a cut-off time of 60 sec.

Light/dark transition test

The apparatus used in this test consisted of a plastic cage (21 cm × 42 cm × 25 cm) that was divided into two equal chambers by a partition containing a door (O'Hara & Co.). One chamber was brightly illuminated (380 ± 20 lux), and the other chamber was dark (0.2 ± 0.1 lux). The offspring was placed in the dark chamber and allowed to move freely between the chambers with the door open for 10 min. The distance traveled (in cm), latency to first enter the lighted chamber (in sec), time spent in each chamber (in sec), and number of transitions were recorded automatically using ImageLD [2].

Open field test

A square acrylic arena (40 cm × 40 cm × 30 cm; Accuscan Instruments, Columbus, OH) was used as the open field. The center of the floor was illuminated at 100 lux using white light. The mouse was placed in one corner of the arena, and the total distance traveled (in cm), vertical activity (i.e., rearing, which was measured by counting the number of photobeam interruptions), time spent in the center of the area (i.e., the middle 20 cm × 20 cm) (in sec), and the number of beam breaks for stereotypic behavior were recorded for 120 min.

Elevated plus maze test

The maze apparatus consisted of two opposing open arms (25 cm × 5 cm), two opposing enclosed arms (25 cm × 5 cm) with 15-cm high transparent walls, and a center square (5 cm × 5 cm) connecting the four arms, all of which were placed at a 90° angles (O'Hara & Co.). The open arms were surrounded by a

raised ledge (3 mm thick and 3 mm high) to prevent the mice from falling off the arms. The arms and center square were composed of white plastic plates and were elevated 55 cm above the floor. For the test, the offspring was placed in the central area facing a closed arm and was allowed to explore the maze for 10 min. The distance traveled (in cm), number of arm entries, entries into open arms (expressed as a percentage), and total time spent in open arms (expressed as a percentage) were calculated automatically using ImageEP [3].

Hot plate test

The offspring was placed on a hot plate (Columbus Instruments, Columbus, OH) set at 55.0 ± 0.3°C, and the latency to the first hind paw response—defined as either a paw lick or a foot shake—was recorded.

Measuring social interactions in a novel environment

Two weight-matched offspring (within 4.6 g of each other) from the same maternal diet group that were previously housed in different cages were placed together in an acrylic box (40 cm × 40 cm × 30cm; O'Hara & Co.) and allowed to explore the box freely for 10 min, and the total duration of contact time (in sec), number of contacts, duration of active contacts (in sec), mean duration per contact (in sec), and distance traveled (in cm) were measured. We defined an "active contact" as when the two mice contacted each other and traveled together for a distance >10 cm. Data acquisition and analysis were performed automatically using ImageSI [4].

Rotarod test

The offspring was placed on a rotating drum (3-cm diameter) on an accelerating rotarod (UGO Basile Accelerating Rotarod, Gemonio, Italy), and the rotational speed of the rotarod increased from 4 rpm to 40 rpm over a 5-min period. The latency to fall off the rotarod (i.e., the duration of time in which the mouse could maintain its balance on the rod) was recorded for 3 trials per day over 2 consecutive days, with a 5-min cut-off time per trial.

Sociability and social novelty preference test

The testing apparatus consisted of a rectangular three-chambered box and a lid fitted with an infrared video camera (O'Hara & Co.). Each chamber was 20 cm × 40 cm × 47 cm, and the walls between the chambers were composed of clear Plexiglas, with small square openings (5 cm × 3 cm) allowing access to each chamber. A small quarter-cylindrical shaped wire cage was placed in the left- and right-side chambers; this wire cage was 21.3 cm in height with a bottom radius of 10.5 cm, with vertical wires spaced 0.5 cm apart. During the habituation session, the subject offspring was placed in the middle chamber and allowed to explore the entire test chamber for 10 min. The next day, a novel male mouse at 9 weeks of age (stranger 1) with no prior contact with the subject was enclosed in either wire cage in the side chambers, which allowed for nose contact but prevented fighting between the mice. The location of stranger 1 in the left and right chambers was systematically alternated between trials. The

subject offspring was then placed in the middle chamber and allowed to explore for 10 min (the sociability test). After the first 10-min test session, a second unfamiliar mouse (stranger 2) was placed in the wire cage on the opposite chamber, and the offspring could choose between the previously unfamiliar mouse (i.e., stranger 1) and the novel unfamiliar mouse (i.e., stranger 2). The time spent each cage and the number of entries around each cage were measured. Data were acquired and analyzed automatically using TimeCSI (O'Hara & Co.).

Startle response/PPI test

Each offspring was placed in a plastic cylinder in the startle reflex measurement system (O'Hara & Co.) and left undisturbed for 10 min in the presence of background white noise (70 dB). After this 10-min period, a startle stimulus (white noise at 110 or 120 dB, applied for 40 msec) was presented to the offspring. To measure PPI, a prepulse sound (74 or 78 dB) was presented 100 msec before the startle stimulus. The startle response to the sound stimulus was recorded for 400 msec from the onset of the startle stimulus. The peak amplitude of the startle amplitude was used as the dependent variable. A test session consisted of six trial types, with two startle stimulus–only trials and four of PPI trials with various combinations of prepulse and startle stimuli (74-110 dB, 78-110 dB, 74-120 dB, and 78-120 dB). Six blocks of these six trial types were presented in a pseudorandom order such that each trial type was

presented once within a block. The mean interval between trials was 15 sec (range 10-20 sec).

Porsolt forced swim test

A transparent plastic cylinder (20 cm height, 10 cm diameter) filled with water (21-23°C) to a height of 12 cm was placed in a white plastic chamber (31 cm × 41 cm × 41 cm; O'Hara & Co.). The offspring was then placed in the cylinder, and the mouse's immobility (measured as a percentage) and distance traveled (in cm) were recorded for 10 min. Specifically, images were captured at 2 frames per sec, and for each pair of successive frames, the area (in pixels) within which the mouse moved was measured. When the area was below a certain threshold, the mouse's behavior was classified as "immobile"; when the area equaled or exceeded that threshold, the mouse was classified as "moving". Periods of immobility lasting less than 2 sec was not included in the analysis. Data were acquired and analyzed automatically using ImagePS [5].

Place recognition test

We used a square (S) chamber (30 cm × 30 cm × 30 cm) composed of transparent plastic with steel grids on the floor, as well as a circular (C) chamber (30 cm height, 34 cm diameter) composed of white plastic. During the learning session, the offspring was placed in chamber S and allowed to freely explore for 6 min; 24 hours after the learning session, the offspring was placed in either the

experienced chamber (S) or the novel chamber (C) and allowed to explore for 3 min. Data were acquired and analyzed automatically using ImageOF [6].

Object location test

The offspring was placed in one corner of an open field apparatus (40 cm × 40 cm × 30 cm), with the center of the apparatus illuminated at 100 lux. On days 1 through 3, the offspring was allowed to explore the chamber for 10 min for habituation. On day 4, two identical objects were placed 20 cm apart in the upper fifth of the chamber (8 cm from the wall), and the offspring was allowed to explore the chamber for 15 min, serving as the training session. On day 5, one of the two objects was placed in the same location as on day 4, while the other object was placed in a new location in the lower fifth of the chamber, and the offspring was again allowed to explore the chamber for 15 min. The time spent exploring the object located in the novel location and the total time spent exploring both objects were measured.

T-maze spontaneous alternation test

We used a modified automated T-maze apparatus (O'Hara & Co.) [6]. The apparatus was constructed of white plastic runways with 25-cm high walls, partitioned into the following 6 areas: the stem of the T, a straight runway, the left and right arms, and passageways connecting the arms to the stem of the T. A variety of fixed extra-maze clues surrounded the apparatus. For this test, the offspring was subjected to a session consisting of 10 trials per day for five

consecutive days (with a cut-off time of 50 min). A correct response in each lap indicated that the offspring chose the opposite direction from the last lap. Data were acquired and analyzed automatically using ImageTM [6].

Barnes maze test

Barnes circular maze consisted of a 100-cm diameter white acrylic platform with 12 holes spaced evenly along the perimeter at a height of 80 cm (O'Hara & Co.). A black escape box (17 cm × 13 cm × 7 cm) was placed under one of the holes to allow the offspring to enter the box. The location of the escape box was unchanged during a given offspring's test, but was randomized among the offspring. The maze was rotated daily to prevent any bias based on olfactory and/or proximal cues within the maze. Three trials per day were conducted for 5 consecutive days (excluding weekends) as a training session. On day 6, a probe trial was conducted without the escape box to confirm that this spatial task was acquired based on navigation using distal environmental cues. Another probe trial was conducted 30 days after the last training day in order to measure spatial remote memory, and the time spent at each hole was recorded. Data were acquired and analyzed automatically using ImageBM [7].

Measurement of home cage activity

We used a system that automatically analyzed the offspring's locomotor activity [8]. The system contains a home cage (25 cm × 15 cm × 23.5 cm, interior dimensions), a filtered cage top, and an infrared video camera attached to the

top of a stand. Each offspring was individually housed in its home cage, and its locomotor activity was monitored for two weeks. The output from the video camera was fed into a computer, and images from each cage were captured at a rate of one frame per second; the distance traveled was measured automatically using ImageHA, and the mean values averaged for each hour from 13:00 on day 3 through 12:00 on day 6 12:00 (i.e., the 1st week) and each hour from 13:00 on day 11 through 12:00 on day 13 (i.e., the 2nd week) were used for statistical analysis.

Measurement of voluntary running activity

We used a single running wheel (ENV-044, Med Associates, Fairfax, VT) placed in the home cage after measuring home cage activity. Each offspring was individually housed in its home cage with a running wheel, and wheel-running activity was measured for 2-3 weeks using a USB interface hub (DIG-804, Med Associates) which relayed data to Wheel Manager Software (SOF-860, Med Associates); the collected data were analyzed using Wheel Analysis Software (SOF-861, Med Associates). The mean values averaged for each hour from 13:00 on day 3 through 12:00 on day 6 (i.e., the 1st week) and from 13:00 on day 11 through 12:00 on day 13 (i.e., the 2nd week) were used for statistical analysis.

Fear conditioning

The offspring was placed in a conditioning chamber (26 cm × 34 cm × 29 cm, 100 lux illumination) containing a stainless-steel grid floor (O'Hara & Co.). The offspring was first allowed to explore freely for 2 min, after which the conditioned stimulus (CS, 55 dB white noise) was applied for 30 sec with the unconditioned stimulus (US, a 0.3-mA foot shock) applied during the last 2 sec of the CS. The CS-US pair was presented three times with a 2-min interval. One day after the conditioning session, the offspring was placed into the same conditioning chamber for 5 min (the context test). Next, a cued test in an altered context was performed using a triangular box (35 cm × 35 cm × 40 cm) composed of white opaque plastic, which was located in a different sound-attenuated room. After a 3-min free-moving period in the triangular chamber, the CS (55 dB white noise) was applied for 180 seconds. The context and cued tests were then repeated 28 days after the conditioning session in order to measure memory retention. Data acquisition, control of the stimuli, and data analysis were performed automatically using ImageFZ [9].

Social interaction test in the home cage

The system for monitoring social interactions consisted of a home cage (25 cm × 15 cm × 23.5 cm, interior dimensions) and a cage top fitted with an infrared video camera. Two offspring from the same maternal diet group that had been housed separately were placed together in the home cage, and video images in each cage were captured at a rate of 1 frame per second. Social interaction was measured by counting the number of animals detected in each frame. We also

measured the activity level of the offspring by quantifying the number of pixels that changed between each pair of successive frames. The mean number of animals and the total activity level in each 1-min bin were automatically calculated for 1 week using ImageHA [8], and the mean values averaged for each hour from 13:00 on day 3 through 12:00 on day 6 were used for statistical analysis.

SUPPLEMENTAL RESULTS

Consuming the LA^{high}/ALA^{low} diet during pregnancy does not affect USVs in early postnatal offspring

We examined social communication behaviors in offspring at postnatal day 7 by measuring maternal separation–induced USVs, which is generally considered to represent the pup's call for its mother [10]. We found no difference in the number, latency, duration, or frequency of USVs between control and LA^{high}/ALA^{low} offspring, regardless of sex (**Supplemental Figure 3**). These data indicate that maternal consumption of the LA^{high}/ALA^{low} diet during pregnancy does not affect the offspring's early social communication behaviors.

In utero exposure to the LA^{high}/ALA^{low} diet affects wire hang performance in adult female offspring

Next, we compared physical characteristics between control and LA^{high}/ALA^{low} offspring at 11-13 weeks of age and found no significant difference in the righting reflex, whisker twitch reflex, or ear twitch reflex between groups, regardless of sex (data not shown). In addition, we found no significant difference in body weight, body temperature, grip strength, wire hang latency, or sensitivity to a painful stimulus between control and LA^{high}/ALA^{low} offspring, with the sole exception of a significant difference in wire hang latency in the female offspring (**Supplemental Figure 4A-E**). Thus, *in utero* exposure to the LA^{high}/ALA^{low} diet appears to cause an alteration in some facets of motor and/or muscle function specifically in female offspring.

We next measured motor coordination in the offspring at 12-13 weeks of age for male offspring and at 13-14 weeks of age for female offspring using the rotarod test, but found no difference in the latency to fall between control and LA^{high}/ALA^{low} offspring, regardless of sex (**Supplemental Figure 4F**). Thus, *in utero* exposure to the LA^{high}/ALA^{low} diet does not appear to affect the offspring's coordinated motor function.

In utero exposure to the LA^{high}/ALA^{low} diet does not affect anxiety-related behaviors in adulthood

Next, we assessed whether *in utero* exposure to the LA^{high}/ALA^{low} diet has long-term effects on locomotor activity and/or anxiety-related behaviors using the light/dark transition test and the elevated plus maze test at 11-13 weeks of age. These behavioral tests were performed using devices that were not familiar to the offspring and therefore reflected behaviors observed in a novel environment. Based on the light/dark transition test, we found no significant difference between groups with respect to three aspects of anxiety-related behaviors—namely, time spent in the light chamber (**Supplemental Figure 5A**), the number of transitions between chambers (**Supplemental Figure 5B**), the latency to first enter the light chamber (**Supplemental Figure 5C**)—or the distance traveled in each chamber (**Supplemental Figure 5D**), again regardless of sex. Using the elevated plus maze test as an additional measure of anxiety-related behaviors, we also found no significant difference between groups with respect to time spent in the open arms (**Supplemental Figure 5E**), the total number of entries

into the open and closed arms (**Supplemental Figure 5F**), the relative number of entries into the open arms (**Supplemental Figure 5G**), or total distance traveled (**Supplemental Figure 5H**). These results suggest that adult mice that were exposed *in utero* to the LA^{high}/ALA^{low} diet do not exhibit higher anxiety-related behaviors in several types of novel environments.

***In utero* exposure to the LA^{high}/ALA^{low} diet does not affect sensorimotor gating in adulthood**

Sensorimotor gating is a brain process by which the brain filters irrelevant and uninformative sensory inputs in order to prevent sensory overload [11]. This important process can be measured using a PPI protocol in which a preceding low-intensity stimulus suppresses the response to a subsequent stronger startle stimulus. We therefore measured sensorimotor gating using the acoustic startle response test and a PPI protocol at 14-15 weeks of age for male offspring and at 15-16 weeks of age for female offspring and found no significant difference between control and LA^{high}/ALA^{low} offspring with respect to the acoustic startle response to either 110 or 120 dB stimuli (**Supplemental Figure 6A**) or PPI induced by a 74 or 78 dB prepulse (**Supplemental Figure 6B**), regardless of sex. Thus, *in utero* exposure to the LA^{high}/ALA^{low} diet does not appear to affect sensorimotor gating in adulthood.

***In utero* exposure to the LA^{high}/ALA^{low} diet does not induce apparent alterations in depressive behavior in adulthood**

Next, we measured depressive behavior in the offspring at 15-16 weeks of age using the Porsolt forced swim test. We found that the time during which the mouse was immobile was lower in LA^{high}/ALA^{low} offspring compared to control offspring on the first day of the test, with a stronger effect observed in the female offspring (**Supplemental Figure 7A**); on the second day, however, we found no significant difference between control and LA^{high}/ALA^{low} offspring, regardless of sex (**Supplemental Figure 7B**). We also found no significant difference between groups with respect to the distance that the mice swam, regardless of sex and the day tested (**Supplemental Figure 7CD**). These results suggest that *in utero* exposure to the LA^{high}/ALA^{low} diet does not have an apparent effect on depressive behavior in adulthood.

***In utero* exposure to the LA^{high}/ALA^{low} diet does not affect pattern separation in adulthood**

Pattern separation is a memory process by which the animal can form separate representations from highly similar stimuli [12]. We therefore examined whether *in utero* exposure to the LA^{high}/ALA^{low} diet affects this process using the place recognition test at 17-18 weeks of age. We found no significant difference between chambers with respect to the distance traveled (i.e., the distance explored in the chamber) on the first day, regardless of diet and sex (**Supplemental Figure 8A**). On the second day, both control and LA^{high}/ALA^{low} offspring—of both sexes—explored a larger distance in the chamber containing a different combination than in the chamber containing the same combination

(**Supplemental Figure 8B**). Finally, we found no significant difference between control and LA^{high}/ALA^{low} offspring with respect to the ratio between of the distance traveled on the second day and the distance traveled on the first day, regardless of sex (**Supplemental Figure 8C**). Thus, *in utero* exposure to the LA^{high}/ALA^{low} diet does not appear to affect pattern separation measured using the place recognition test.

In utero **exposure to the LA^{high}/ALA^{low} does not affect spatial working memory in adulthood**

Next, we examined whether *in utero* exposure to the LA^{high}/ALA^{low} diet affects spatial working memory using the T-maze spontaneous alternation test at 19-20 weeks of age for male offspring and at 20-21 weeks of age for female offspring, but found no significant difference between groups with respect the percentage of correct responses (**Supplemental Figure 9A**), the latency to complete the task (**Supplemental Figure 9B**), or distance traveled (**Supplemental Figure 9C**), regardless of sex. Thus, *in utero* exposure to the LA^{high}/ALA^{low} diet does not appear to affect spatial working memory in adulthood.

In utero **exposure to the LA^{high}/ALA^{low} diet does not affect fear memory in adulthood**

Finally, we examined fear memory using the fear conditioning test at 55-56 weeks of age, but found no significant difference between LA^{high}/ALA^{low} and control offspring difference with respect to the rate of freezing (**Supplemental**

Figure 10A) or the distance traveled (**Supplemental Figure 10B**) during the conditioning session, regardless of their sex, suggesting no changes in fear conditioning.

We then examined fear memory retention using the contextual and cued fear conditioning tests performed one day (**Supplemental Figure 11A-D**) and 4 weeks (**Supplemental Figure 11E-H**) after the conditioning session (i.e., we examined offspring at 55-56 weeks of age and at 59-60 weeks of age, respectively), but again found no significant difference between the LA^{high}/ALA^{low} offspring and their sex-matched controls. Thus, *in utero* exposure to the LA^{high}/ALA^{low} diet does not appear to affect fear memory retention in adulthood.

SUPPLEMENTAL REFERENCES

1. R.O. Tachibana, K. Kanno, S. Okabe, K.I. Kobayasi, K. Okanoya, USVSEG: A robust method for segmentation of ultrasonic vocalizations in rodents, PLoS One 15 (2) (2020) e0228907, https://doi.org/10.1371/journal.pone.0228907.
2. K. Takao, T. Miyakawa, Light/dark transition test for mice, J. Vis. Exp. 1 (2006) 104, https://doi.org/10.3791/104.
3. M. Komada, K. Takao, T. Miyakawa, Elevated plus maze for mice, J. Vis. Exp. 22 (2008), https://doi.org/10.3791/1088.
4. H. Shoji, K. Takao, S. Hattori, T. Miyakawa, Age-related changes in behavior in C57BL/6J mice from young adulthood to middle age, Mol. Brain 9 (2016) 11, https://doi.org/10.1186/s13041-016-0191-9.
5. K. Fujii, H. Otofuji, Y. Nakamura, K. Y., M. Adachi, E. Sasakawa, et al., Comprehensive behavioral analysis of mice repeatedly treated with propofol, Transl. Regul. Sci. 1 (2) (2019) 46-57, https://doi.org/10.33611/trs.1_46.
6. R. Ohashi, K. Takao, T. Miyakawa, N. Shiina, Comprehensive behavioral analysis of RNG105 (Caprin1) heterozygous mice: Reduced social interaction and attenuated response to novelty, Sci. Rep. 6 (2016) 20775, https://doi.org/10.1038/srep20775.
7. M. Yasumura, T. Yoshida, M. Yamazaki, M. Abe, R. Natsume, K. Kanno, et al., IL1RAPL1 knockout mice show spine density decrease, learning deficiency, hyperactivity and reduced anxiety-like behaviours, Sci. Rep. 4 (2014) 6613, https://doi.org/10.1038/srep06613.

8. H. Shoji, Y. Irino, M. Yoshida, T. Miyakawa, Behavioral effects of long-term oral administration of aluminum ammonium sulfate in male and female C57BL/6J mice, Neuropsychopharmacol. Rep. 38 (1) (2018) 18-36, https://doi.org/10.1002/npr2.12002.
9. H. Shoji, K. Takao, S. Hattori, T. Miyakawa, Contextual and cued fear conditioning test using a video analyzing system in mice, J. Vis. Exp. 85 (2014), https://doi.org/10.3791/50871.
10. M. Iijima, S. Chaki, Separation-induced ultrasonic vocalization in rat pups: further pharmacological characterization, Pharmacol. Biochem. Behav. 82 (4) (2005) 652-657, https://doi.org/10.1016/j.pbb.2005.11.005.
11. N.R. Swerdlow, M.A. Geyer, Using an animal model of deficient sensorimotor gating to study the pathophysiology and new treatments of schizophrenia, Schizophr. Bull 24 (2) (1998) 285-301, https://doi.org/10.1093/oxfordjournals.schbul.a033326.
12. W. Deng, J.B. Aimone, F.H. Gage, New neurons and new memories: how does adult hippocampal neurogenesis affect learning and memory?, Nat. Rev. Neurosci. 11 (5) (2010) 339-350, https://doi.org/10.1038/nrn2822.

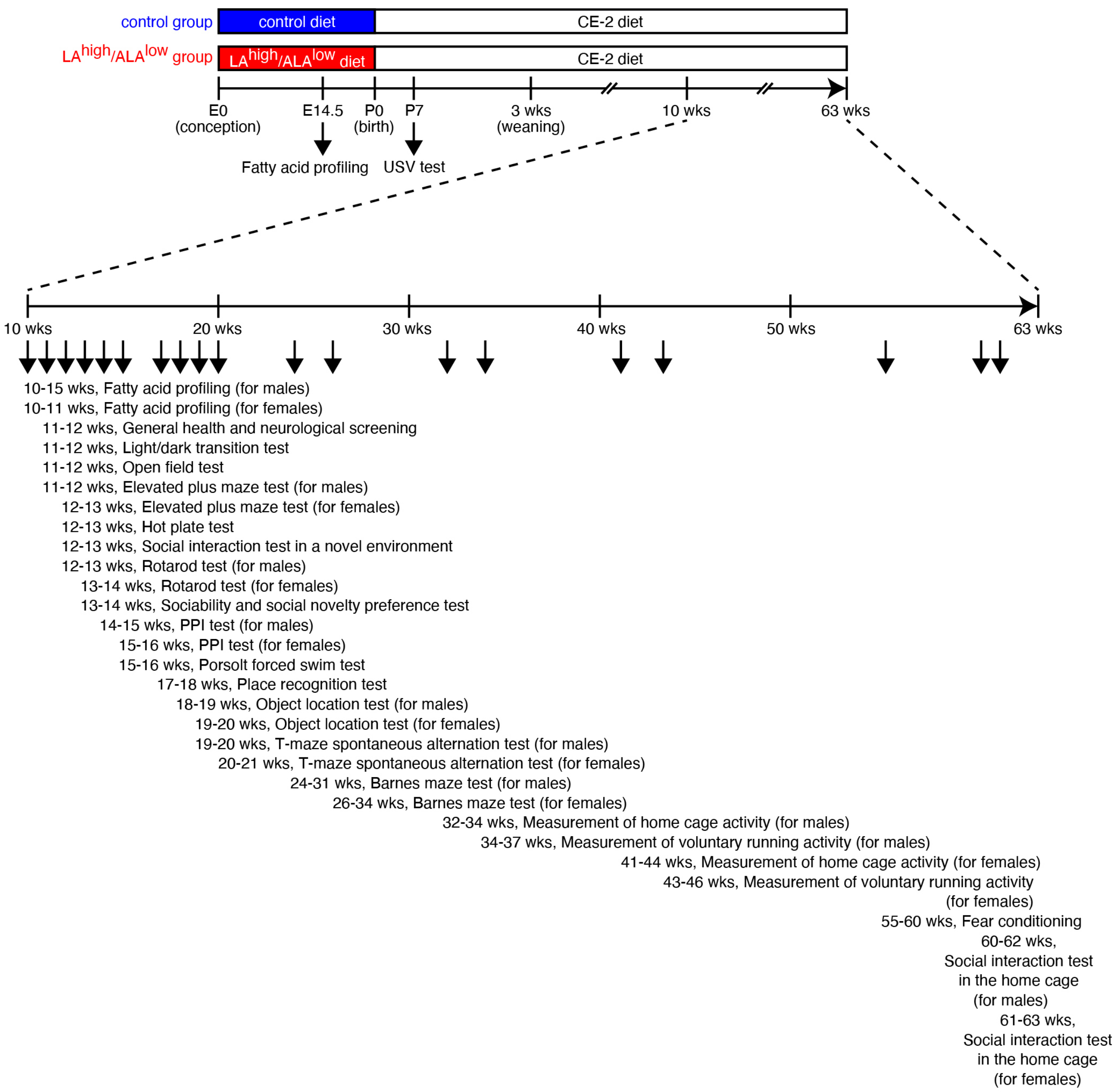

Supplemental Figure 1

The experimental outline. ALA, α-linolenic acid; LA, linoleic acid; PPI, prepulse inhibition; P0, postnatal day 0; P7, postnatal day 7; USV, ultrasonic vocalization; wks, weeks of age.

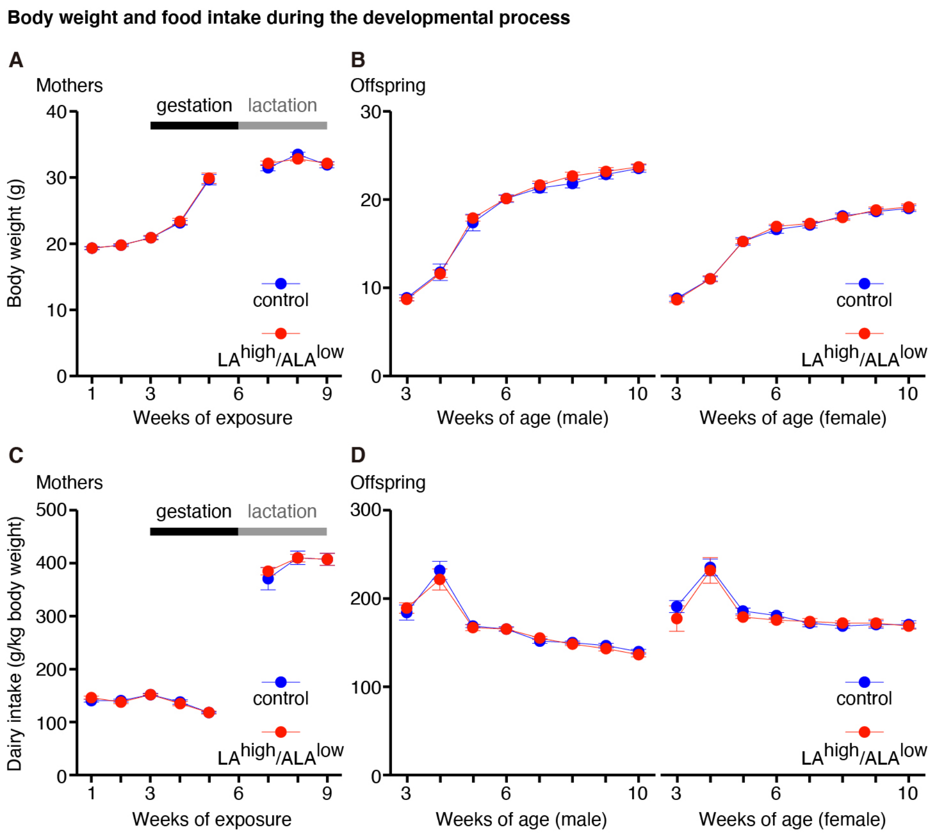

Supplemental Figure 2

Developmental outcomes of mothers fed the control or LA^{high}/ALA^{low} diet during gestation, and their offspring. **A-B**, Time course of body weight measured in mothers (**A**) and offspring (**B**) (n = 7-8/group). Data were analyzed using a two-way ANOVA (with week as a repeated measure; **A**) or a three-way ANOVA (with age in weeks as a repeated measure; **B**). **C-D**, Time course of daily food intake measured in the mothers (**C**) and offspring (**D**) (n = 7-8/group). Data were analyzed using a two-way ANOVA (with week as a repeated measure; **C**) or a three-way ANOVA (with age in weeks as a repeated measure; **D**). ALA, α-linolenic acid; LA, linoleic acid.

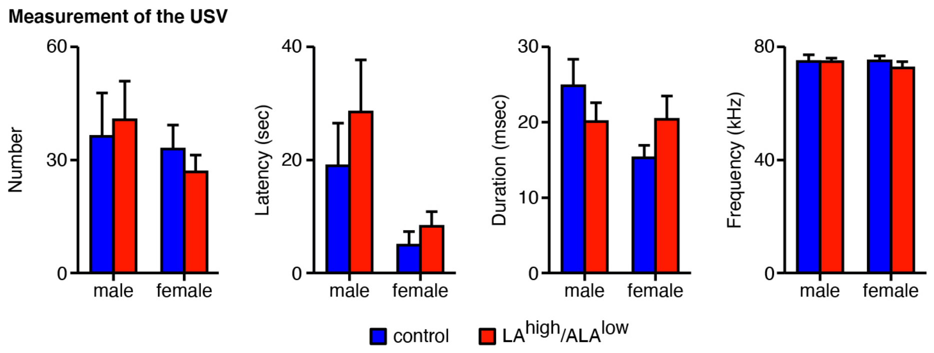

Supplemental Figure 3

USVs measured in offspring exposed *in utero* to the control or LA^{high}/ALA^{low} diet. Summary of the number, latency, duration, and frequency of USVs measured in the offspring (n = 10-14/group). Data were analyzed using a two-way ANOVA. ALA, α-linolenic acid; LA, linoleic acid; USV, ultrasonic vocalization.

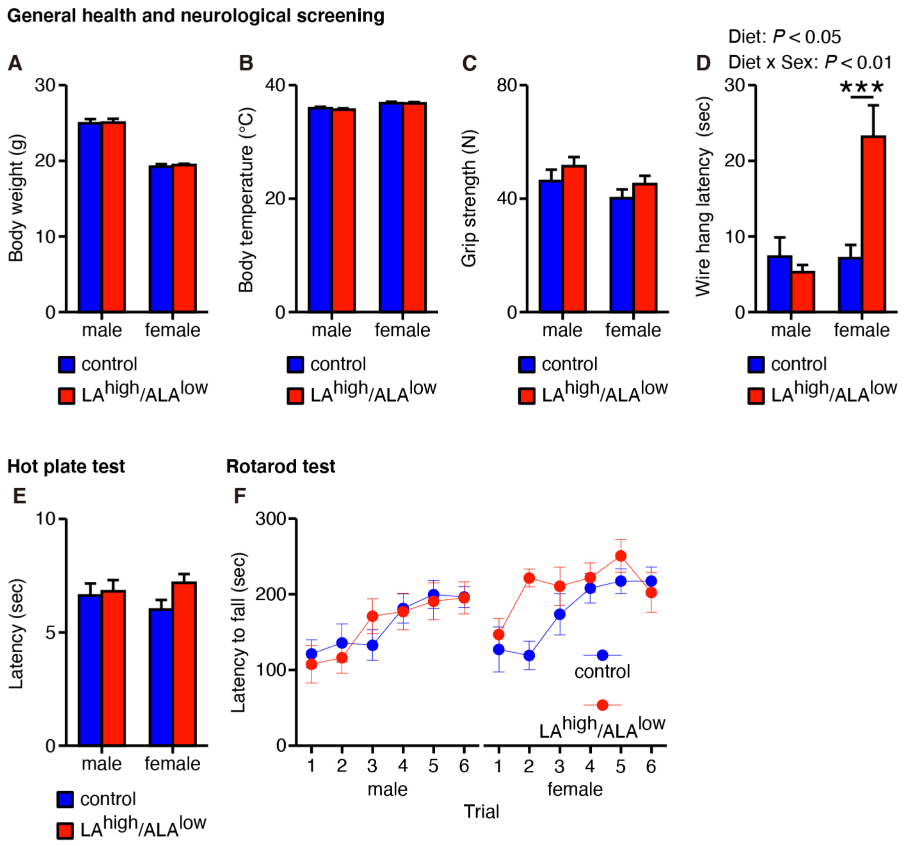

Supplemental Figure 4

Physical characteristics and motor function measured in offspring exposed *in utero* exposed to the control or LA^{high}/ALA^{low} diet. **A-D**, Summary of body weight (**A**), body temperature (**B**), grip strength (**C**), and wire hang latency (**D**) measured in adult offspring (n = 8-11/group). Data were analyzed using a two-way ANOVA with post hoc simple main effect analysis for multiple comparisons. **E**, Summary of the latency to the first paw response in the hot plate test measured in adult offspring (n = 8-11/group). Data were analyzed using a two-way ANOVA. **F**, Summary of the time to fall from a rotating rotarod measured in adult offspring (n = 8-11/group). Data were analyzed using a three-way ANOVA (trial as repeated measure). ***P < 0.001 (simple main effect analysis). ALA, α-linolenic acid; LA, linoleic acid.

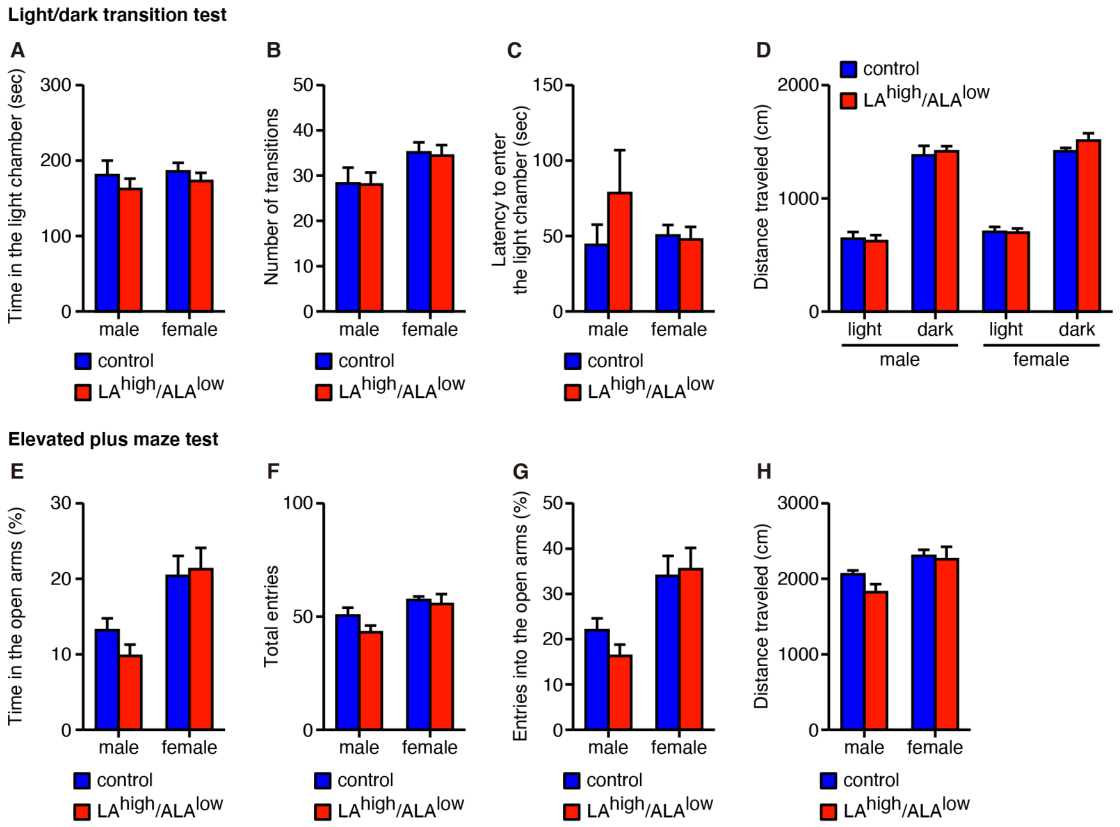

Supplemental Figure 5

Locomotor activity and anxiety-related behaviors measured in offspring exposed *in utero* to the control or LA^{high}/ALA^{low} diet. **A-D**, Summary of time spent in the light chamber (**A**), the number of transitions (**B**), the latency to enter the light chamber (**C**), and the distance traveled in each chamber (**D**) in the light/dark transition test measured in adult offspring (*n* = 8-11/group). **E-H**, Summary of time spent in the open arms (**E**), the number of total entries into the open and closed arms (**F**), the relative number of entries into the open arms (**G**), and the total distance traveled (**H**) in the elevated plus maze test measured in adult offspring (*n* = 7-11/group). Data were analyzed using a two-way ANOVA. ALA, α-linolenic acid; LA, linoleic acid.

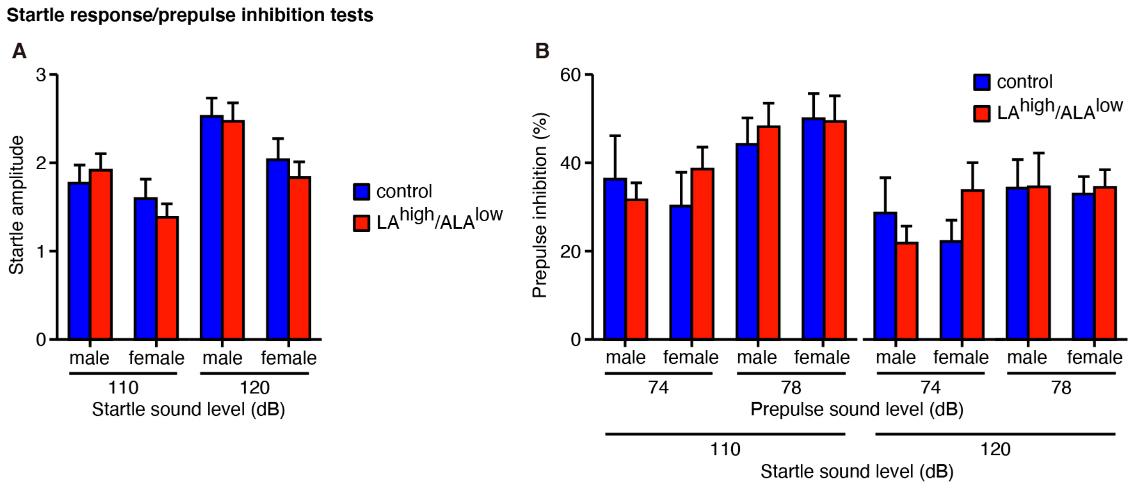

Supplemental Figure 6

Sensorimotor gating measured in offspring exposed *in utero* to the control or LA^{high}/ALA^{low} diet. **A**, Summary of startle amplitude measured using the startle response test in adult offspring (*n* = 8-11/group). **B**, Summary of prepulse inhibition measured in adult offspring (*n* = 8-11/group). Data were analyzed using a two-way ANOVA. ALA, α-linolenic acid; LA, linoleic acid.

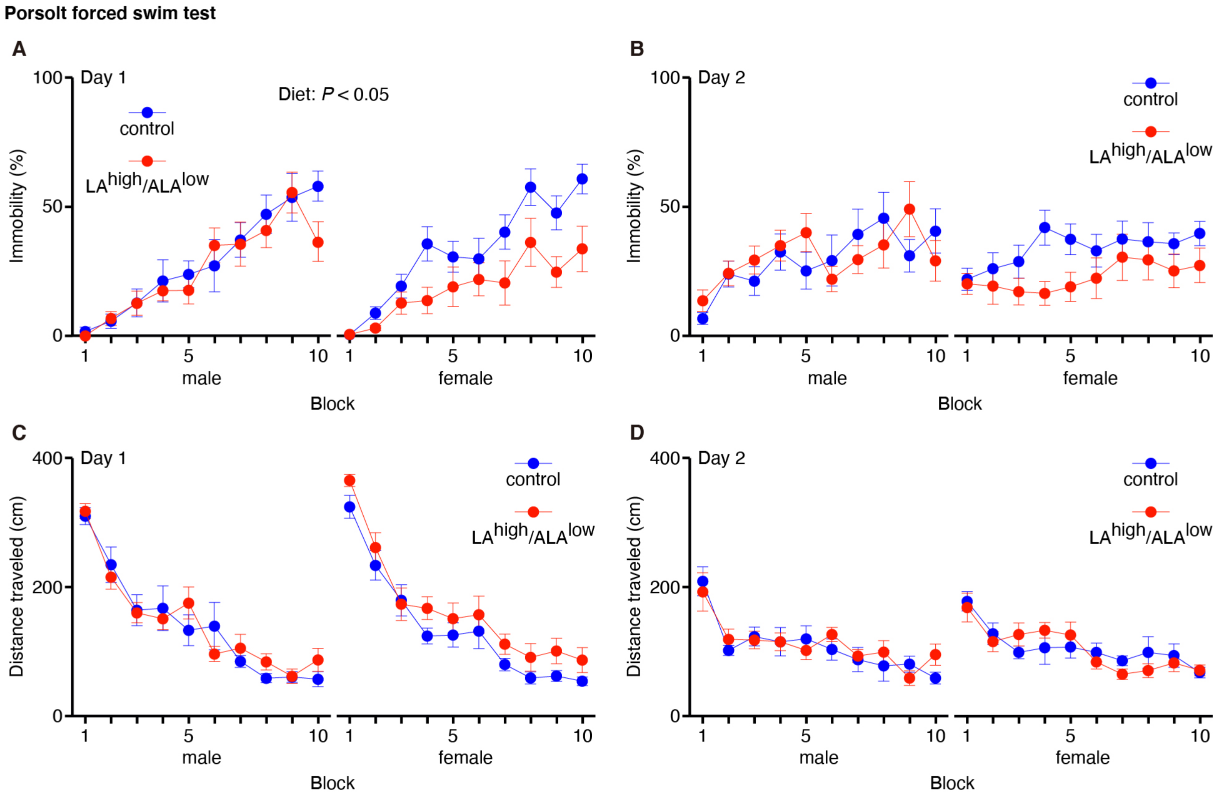

Supplemental Figure 7

Depressive behavior measured in offspring exposed *in utero* to the control or LA^{high}/ALA^{low} diet. **A-B**, Percentage of time the mouse was immobile in the Porsolt forced swim test measured on the first day (**A**) and the second day (**B**) in adult offspring (n = 8-11/group). **C-D**, Distance traveled in the Porsolt forced swim test measured on the first day (**C**) and the second day (**D**) in adult offspring (n = 8-11/group). In **A-D**, data were analyzed using a three-way ANOVA (with block as a repeated measure). ALA, α-linolenic acid; LA, linoleic acid.

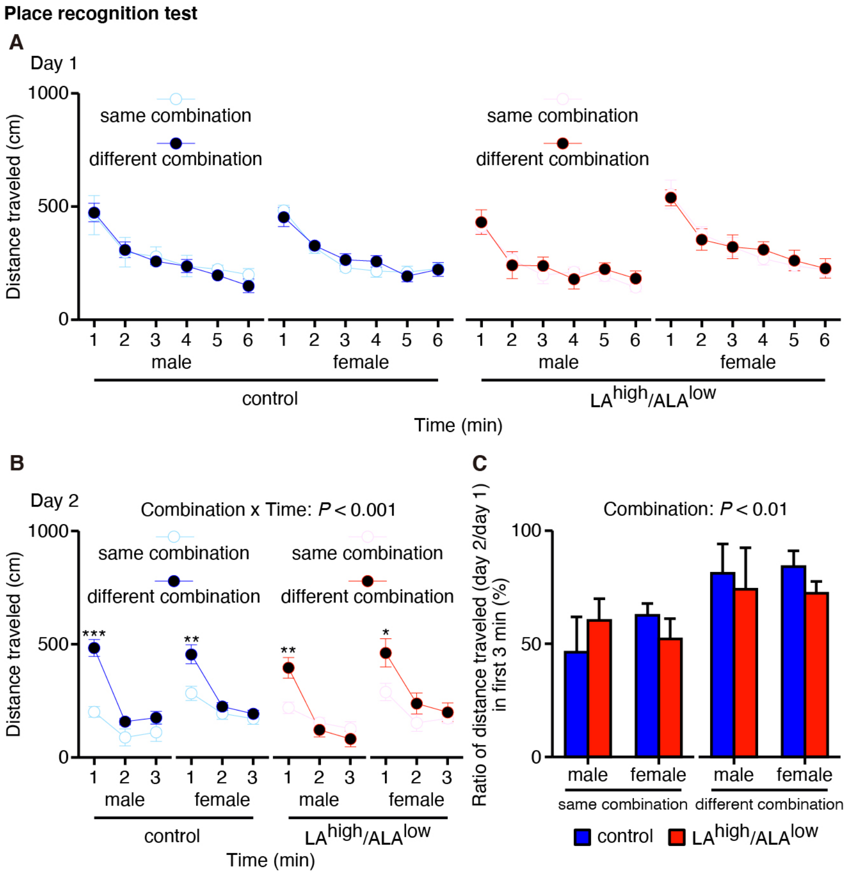

Supplemental Figure 8

Pattern separation measured in offspring exposed *in utero* to the control or LA^{high}/ALA^{low} diet. **A-B**, Distance traveled on the first day (**A**) and the second day (**B**) in the place recognition test (*n* = 4-6/group). Data were analyzed using a three-way ANOVA (with time as a repeated measure) with post hoc simple main effect analysis for multiple comparisons. **C**, Summary of the ratio between the distance traveled on the second day and the distance traveled on the first day, measure in the first 3 min of the test (*n* = 4-6/group). Data were analyzed using a three-way ANOVA. **P* < 0.05, ***P* < 0.01, ****P* < 0.001 (simple main effect analysis). ALA, α-linolenic acid; LA, linoleic acid.

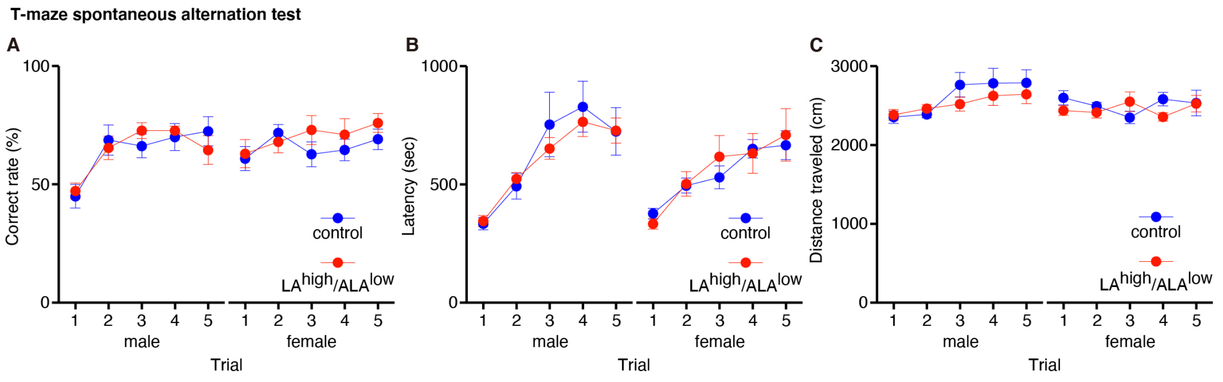

Supplemental Figure 9

Spatial working memory measured in offspring exposed *in utero* to the control or LA^{high}/ALA^{low} diet. **A-C**, The percentage of correct tasks performed (**A**), latency to complete the task (**B**), and distance traveled (**C**) measured using the T-maze spontaneous alternation test (*n* = 8-11/group). Data were analyzed using a three-way ANOVA (with trial as a repeated measure). ALA, α-linolenic acid; LA, linoleic acid.

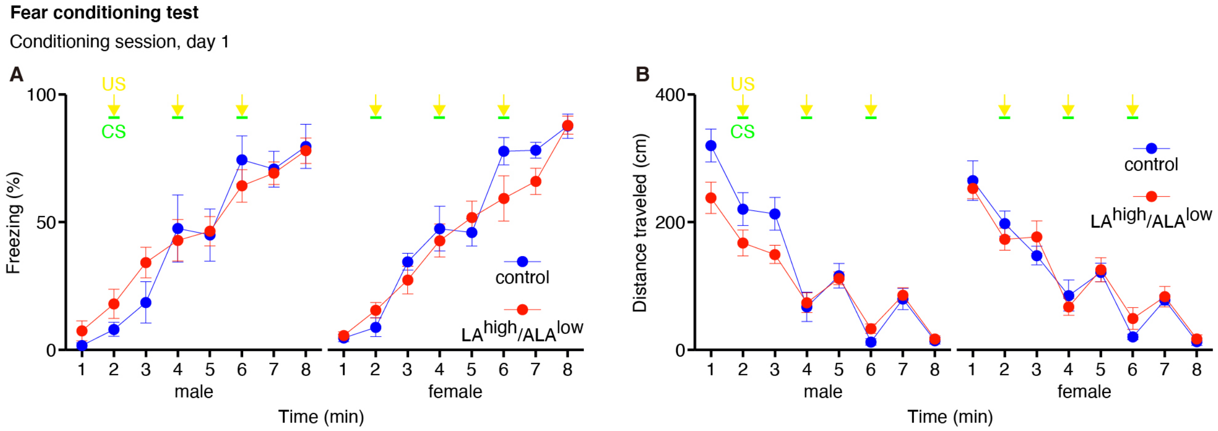

Supplemental Figure 10

Fear conditioning measured in offspring exposed *in utero* to the control or LA^{high}/ALA^{low} diet. Food shock was used as the unconditioned stimulus (US), and an audio tone was used as the conditioned stimulus (CS). **A-B**, Percentage of freezing (**A**) and distance traveled (**B**) measured during the fear conditioning test (*n* = 6-11/group). Data were analyzed using a three-way ANOVA (with time as a repeated measure). ALA, α-linolenic acid; LA, linoleic acid.

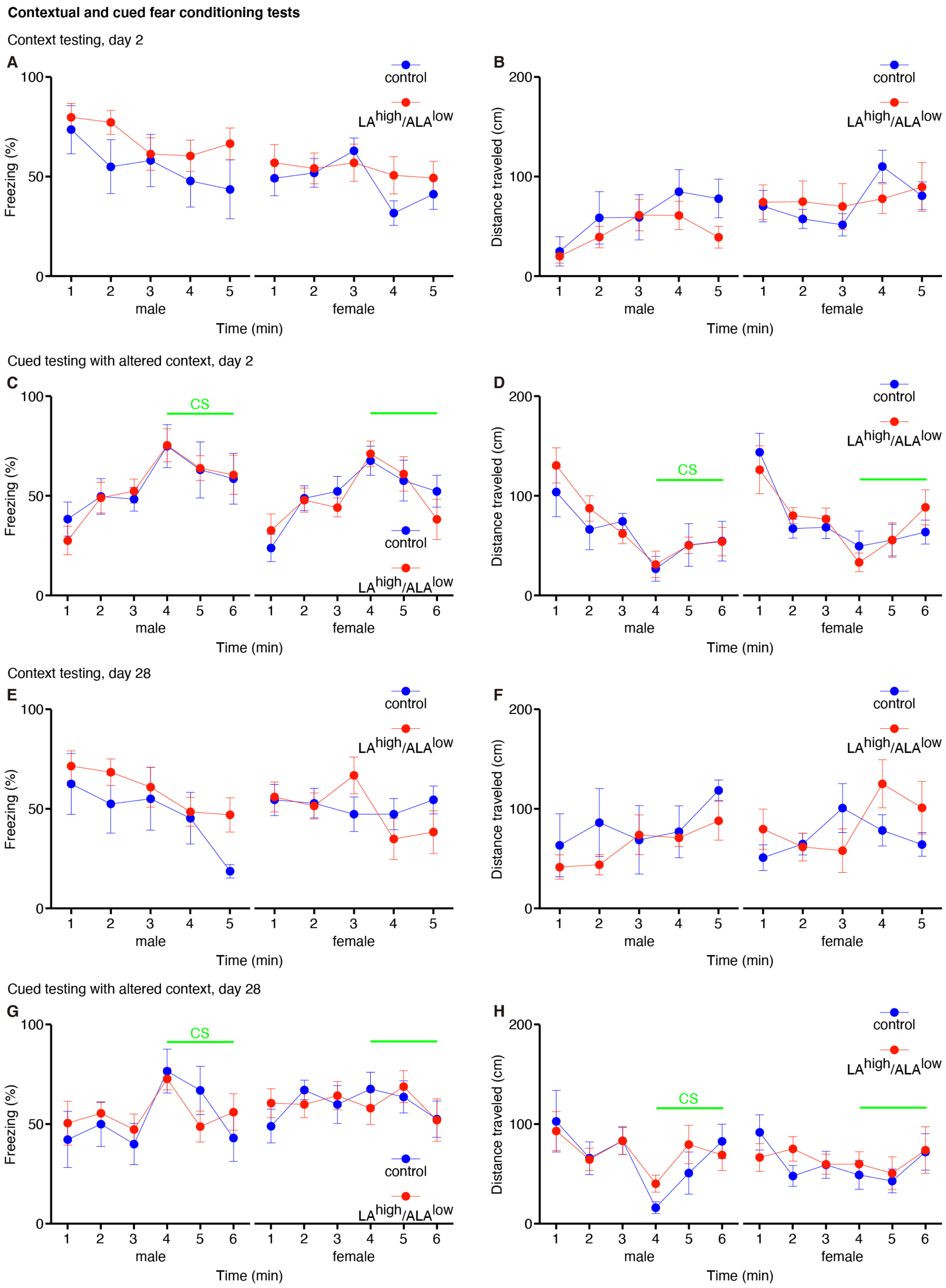

Supplemental Figure 11

Fear memory retention measured in offspring exposed *in utero* to the control or

LA^{high}/ALA^{low} diet. An audio tone was used as the conditioned stimulus (CS). **A-B**, Percentage of freezing events (**A**) and distance traveled (**B**) measured during the contextual fear conditioning test performed one day after fear conditioning (*n* = 6-11/group). **C-D**, Percentage of freezing (**C**) and distance traveled (**D**) measured during the cued fear conditioning test performed one day after fear conditioning (*n* = 6-11/group). **E-F**, Percentage of freezing (**E**) and distance traveled (**F**) measured during the contextual fear conditioning test performed 28 days after fear conditioning (*n* = 6-11/group). **G-H**, Percentage of freezing (**G**) and distance traveled (**H**) measured during the cued fear conditioning test performed 28 days after fear conditioning (*n* = 6-11/group). All data were analyzed using a three-way ANOVA (with time as a repeated measure). ALA, α-linolenic acid; LA, linoleic acid.

Supplemental Table 1. Excluded mice from the behavioral analyses.

Figure	Data index	Excluded mice (reason)
Figure 1F	Distance traveled (2nd week)	the male control group, *n* = 1 (died before the test)
Figure 1G	Number of rotations (1st week)	the male control group, *n* = 2 (died before the test)<br>the female control group, *n* = 5 (technical problems with the video-analysis system)<br>the female LA^{high}/ALA^{low} group, *n* = 4 (technical problems with the video-analysis system)
Figure 1H	Number of rotations (2nd week)	the male control group, *n* = 2 (died before the test)<br>the male LA^{high}/ALA^{low} group, *n* = 2 (machine trouble)
Figure 2N-O	Number of particles<br>Activity level	the male control group, *n* = 2 (died before the test)<br>the female control group, *n* = 1 (died before the test)
Supplemental Figure 5E-H	Time in the open arms<br>Total entries<br>Entries into the open arms<br>Distance traveled	the male control group, *n* = 1 (dropped from the open arm)
Supplemental Figure 10A-B	Freezing<br>Distance traveled	the male control group, *n* = 2 (died before the test)<br>the female control group, *n* = 1 (died before the test)
Supplemental Figure 11A-H	Freezing<br>Distance traveled	the male control group, *n* = 2 (died before the test)<br>the female control group, *n* = 1 (died before the test)

ALA, α-linolenic acid; LA, linoleic acid.

Supplemental Table 3. Brain fatty acid profiles measured in adult offspring exposed *in utero* to the control or LA^{high}/ALA^{low} diet. $n = 3$/group. Data were analyzed using a two-way ANOVA. Fatty acids that contribute to >1% of total fatty acids in either the embryonic or adult brain are shown (see also Table 3).

Fatty acid	control male offspring	LA^{high}/ALA^{low} male offspring	control female offspring	LA^{high}/ALA^{low} female offspring
14:0	0.2% ± 0.0%	0.3% ± 0.1%	0.2% ± 0.0%	0.3% ± 0.0%
16:0	23.4% ± 0.1%	23.3% ± 0.1%	23.3% ± 0.1%	23.2% ± 0.0%
18:0[2]	22.9% ± 0.1%	22.5% ± 0.1%	22.6% ± 0.1%	23.0% ± 0.3%
20:0	1.9% ± 0.1%	1.9% ± 0.0%	1.8% ± 0.1%	1.7% ± 0.1%
16:1	0.7% ± 0.0%	0.6% ± 0.0%	0.6% ± 0.0%	0.6% ± 0.0%
18:1*n*-7[1]	4.1% ± 0.0%	3.9% ± 0.0%	4.0% ± 0.0%	3.9% ± 0.0%
18:1*n*-9	18.3% ± 0.3%	18.2% ± 0.2%	17.9% ± 0.2%	17.7% ± 0.1%
24:1	1.5% ± 0.1%	1.6% ± 0.1%	1.5% ± 0.1%	1.4% ± 0.2%
18:2*n*-6	1.0% ± 0.0%	0.9% ± 0.1%	0.8% ± 0.0%	0.7% ± 0.0%
20:4*n*-6	8.6% ± 0.1%	8.5% ± 0.1%	8.9% ± 0.1%	8.8% ± 0.1%
22:4*n*-6[1]	1.8% ± 0.0%	1.9% ± 0.0%	1.9% ± 0.0%	2.0% ± 0.0%
22:5*n*-6	0.1% ± 0.0%	0.1% ± 0.0%	0.1% ± 0.0%	0.2% ± 0.0%
22:6*n*-3	13.4% ± 0.4%	14.0% ± 0.2%	14.3% ± 0.2%	14.4% ± 0.0%
total SFAs[2]	48.8% ± 0.2%	48.5% ± 0.0%	48.3% ± 0.1%	48.7% ± 0.2%
total MUFAs	25.3% ± 0.4%	25.0% ± 0.3%	24.6% ± 0.3%	24.2% ± 0.1%
total *n*-6 PUFAs	12.3% ± 0.1%	12.3% ± 0.1%	12.6% ± 0.0%	12.4% ± 0.1%
total *n*-3 PUFAs	13.6% ± 0.4%	14.2% ± 0.2%	14.6% ± 0.2%	14.7% ± 0.0%
n-6/*n*-3[1]	0.9 ± 0.0	0.9 ± 0.0	0.9 ± 0.0	0.8 ± 0.0

[1]Diet effect is significant ($P < 0.05$, two-way ANOVA).

[2]Diet x sex interaction is significant ($P < 0.05$, two-way ANOVA), but no difference is detected between the control and LA^{high}/ALA^{low} male offspring or between the control and LA^{high}/ALA^{low} female offspring ($P \geq 0.05$, simple main effect analysis).

ALA, α-linolenic acid; LA, linoleic acid; MUFA, monounsaturated fatty acid; PUFA, polyunsaturated fatty acid; SFA, saturated fatty acid.

Supplemental Table 4. Serum fatty acid profiles measured in mother mice fed the control or LA^{high}/ALA^{low} diet when their embryos were at E14.5. *n* = 3/group. Data were analyzed using a Student's *t*-test, a Welch's *t*-test, or a Mann-Whitney's *U* test. Fatty acids that contribute to >1% of total fatty acids are shown.

Fatty acid	control mother	LA^{high}/ALA^{low} mother
16:0	21.4% ± 0.2%	21.0% ± 0.6%
18:0	11.0% ± 0.7%	11.5% ± 1.4%
16:1	1.2% ± 0.1%[1]	0.7% ± 0.0%[1]
18:1*n*-7	1.4% ± 0.2%	0.7% ± 0.0%
18:1*n*-9	22.3% ± 3.4%[1]	8.5% ± 1.0%[1]
18:2*n*-6	13.9% ± 1.3%	25.5% ± 4.3%
18:3*n*-6	0.5% ± 0.1%	1.4% ± 0.1%
20:4*n*-6	15.2% ± 3.0%	21.7% ± 3.2%
22:5*n*-6	0.0% ± 0.0%[1]	4.9% ± 1.0%[1]
18:3*n*-3	14.1% ± 0.4%	0.1% ± 0.1%
20:5*n*-3	1.2% ± 0.3%[1]	0.0% ± 0.0%[1]
22:6*n*-3	9.8% ± 2.0%	2.8% ± 0.3%
total SFAs	32.9% ± 0.7%	32.9% ± 1.0%
total MUFAs	25.0% ± 3.7%[1]	9.9% ± 1.0%[1]
total *n*-6 PUFAs	29.7% ± 1.9%[2]	54.3% ± 0.3%[2]
total *n*-3 PUFAs	12.5% ± 1.3%[1]	2.8% ± 0.3%[1]
n-6/*n*-3	2.4 ± 0.1[2]	19.5 ± 2.0[2]

[1]There is a significant difference between the control and LA^{high}/ALA^{low} mothers (P < 0.05, unpaired Student's *t*-test).

[2]There is a significant difference between the control and LA^{high}/ALA^{low} mothers (P < 0.05, Welch's *t*-test).

ALA, α-linolenic acid; LA, linoleic acid; MUFA, monounsaturated fatty acid; PUFA, polyunsaturated fatty acid; SFA, saturated fatty acid.

Supplemental Table 5. Liver fatty acid profiles measured in mother mice fed the control or LA^{high}/ALA^{low} diet when their embryos were at E14.5. *n* = 3/group. Data were analyzed using a Student's *t*-test, a Welch's *t*-test, or a Mann-Whitney's *U* test. Fatty acids that contribute to >1% of total fatty acids are shown.

Fatty acid	control mother	LA^{high}/ALA^{low} mother
16:0	23.1% ± 1.0%	23.8% ± 0.6%
18:0	10.2% ± 0.9%	12.2% ± 0.4%
16:1	1.4% ± 0.2%	1.4% ± 0.1%
18:1*n*-7	1.9% ± 0.1%[1]	1.3% ± 0.1%[1]
18:1*n*-9	39.3% ± 3.9%[1]	17.9% ± 0.7%[1]
18:2*n*-6	6.9% ± 0.5%[1]	18.2% ± 0.7%[1]
18:3*n*-6	0.2% ± 0.0%	1.2% ± 0.4%
20:4*n*-6	6.4% ± 0.8%[1]	11.5% ± 0.6%[1]
22:5*n*-6	0.1% ± 0.0%[2]	6.0% ± 0.5%[2]
22:6*n*-3	7.8% ± 1.1%[1]	3.3% ± 0.2%[1]
total SFAs	33.9% ± 1.9%	36.5% ± 0.7%
total MUFAs	43.3% ± 4.2%[1]	20.8% ± 0.8%[1]
total *n*-6 PUFAs	14.0% ± 1.2%[1]	38.2% ± 0.6%[1]
total *n*-3 PUFAs	8.9% ± 1.1%[1]	4.5% ± 1.0%[1]
n-6/*n*-3	1.6 ± 0.1	9.3 ± 2.0

[1]There is a significant difference between the control and LA^{high}/ALA^{low} mothers ($P < 0.05$, unpaired Student's *t*-test).

[2]There is a significant difference between the control and LA^{high}/ALA^{low} mothers ($P < 0.05$, Welch's *t*-test).

ALA, α-linolenic acid; LA, linoleic acid; MUFA, monounsaturated fatty acid; PUFA, polyunsaturated fatty acid; SFA, saturated fatty acid.
